# Mesopelagic N_2_ Fixation Related to Organic Matter Composition in the Solomon and Bismarck Seas (Southwest Pacific)

**DOI:** 10.1371/journal.pone.0143775

**Published:** 2015-12-11

**Authors:** Mar Benavides, Pia H. Moisander, Hugo Berthelot, Thorsten Dittmar, Olivier Grosso, Sophie Bonnet

**Affiliations:** 1 Aix Marseille Université, CNRS/INSU, Université de Toulon, IRD, Mediterranean Institute of Oceanography (MIO) UM 110, 98848, Nouméa, New Caledonia; 2 Department of Biology, University of Massachusetts Dartmouth, 285 Old Westport Road, North Dartmouth, Massachusetts 02747, United States of America; 3 Aix Marseille Université, CNRS/INSU, Université de Toulon, IRD, Mediterranean Institute of Oceanography (MIO) UM 110, 13288, Marseille, France; 4 Research Group for Marine Geochemistry, Institute for Chemistry and Biology of the Marine Environment (ICBM), University of Oldenburg, Carl-von-Ossietzky-Strasse 9–11, D-26129 Oldenburg, Germany; Mount Allison University, CANADA

## Abstract

Dinitrogen (N_2_) fixation was investigated together with organic matter composition in the mesopelagic zone of the Bismarck (Transect 1) and Solomon (Transect 2) Seas (Southwest Pacific). Transparent exopolymer particles (TEP) and the presence of compounds sharing molecular formulae with saturated fatty acids and sugars, as well as dissolved organic matter (DOM) compounds containing nitrogen (N) and phosphorus (P) were higher on Transect 1 than on Transect 2, while oxygen concentrations showed an opposite pattern. N_2_ fixation rates (up to ~1 nmol N L^-1^ d^-1^) were higher in Transect 1 than in Transect 2, and correlated positively with TEP, suggesting a dependence of diazotroph activity on organic matter. The scores of the multivariate ordination of DOM molecular formulae and their relative abundance correlated negatively with bacterial abundances and positively with N_2_ fixation rates, suggesting an active bacterial exploitation of DOM and its use to sustain diazotrophic activity. Sequences of the *nifH* gene clustered with Alpha-, Beta-, Gamma- and Deltaproteobacteria, and included representatives from Clusters I, III and IV. A third of the clone library included sequences close to the potentially anaerobic Cluster III, suggesting that N_2_ fixation was partially supported by presumably particle-attached diazotrophs. Quantitative polymerase chain reaction (qPCR) primer-probe sets were designed for three phylotypes and showed low abundances, with a phylotype within Cluster III at up to 10^3^ *nifH* gene copies L^-1^. These results provide new insights into the ecology of non-cyanobacterial diazotrophs and suggest that organic matter sustains their activity in the mesopelagic ocean.

## Introduction

N_2_ fixation is considered to fuel ~50% of ‘new’ primary production (*sensu* Dugdale et al.; [1]) in oligotrophic oceanic areas, and hence has an important role in modulating the ability of the oceans to sequester carbon dioxide [2]. The amount of fixed N in the oceans depends on the difference between ‘gains’ (N_2_ fixation) and ‘losses’ (denitrification and anaerobic ammonium oxidation -anammox-), which are presently estimated to be unbalanced by ~200 Tg N y^-1^ [3,4]. N_2_ fixation has been classically studied in sunlit oligotrophic tropical and subtropical waters, and only more recently in other nutrient-rich environments such as coastal upwelling areas, oxygen minimum zones (OMZs), and the mesopelagic layer [5,6]. Recent improvements in methodologies and understanding of the marine N cycle raise the question whether extending measurements to higher latitudes and depths would increase N_2_ fixation rates enough to balance fixed N losses [7,8].

Oceanic N_2_ fixation was previously primarily attributed to the filamentous cyanobacterium *Trichodesmium* (e.g. [9]), until the advent of molecular techniques targeting the *nifH* gene revealed that unicellular diazotrophic cyanobacteria are abundant and widespread globally [10,11], and contribute significantly to N_2_ fixation in several oceanic basins [12]. Non-cyanobacterial diazotroph groups (bacteria and archaea) have been detected in numerous studies and across the world’s oceans [13], and their *nifH* sequences represent >80% of the total sequences retrieved from marine samples available in databases [14]. Recent studies have claimed the potentially important diazotrophic activity of non-cyanobacterial diazotrophs in coastal seas like the Baltic Sea (e.g. [15]), as well as oligotrophic open-ocean areas such as the South Pacific [16,17]. Despite their numerical superiority, their N_2_ fixation potential and ecology are largely unknown [14]. While photic autotrophic cyanobacterial diazotrophs require light, P and iron for their activity [18], non-cyanobacterial diazotrophs may exploit a variety of metabolisms for their nutrition, including phototrophy in the sunlit layer [19], chemolithoautotrophy and chemoorganoheterotrophy, which could be also present in aphotic waters [20–22]. Aphotic N_2_ fixation can occur in response to fixed N loss (i.e. in OMZs) in order to balance global fixed N budgets as modeling approaches have suggested [23], and *in situ* data have demonstrated [8,21,24]. Nevertheless, aphotic N_2_ fixation also takes place in fully oxygenated waters [25], presumably in association with particles depleted in oxygen due to intense bacterial respiration [20]. Indeed, N_2_ fixation rates and non-cyanobacterial *nifH* genes have been reported from mesopelagic to abyssopelagic waters [21,22,26], but the factors controlling their activity and diversity, as well as their metabolism are currently not understood (i.e. [20]).

In order to gain new insights into the ecology of aphotic N_2_ fixation, in this study we investigate the potential connections between non-cyanobacterial mesopelagic N_2_ fixation and *in situ* organic matter (chemoorganoheterotrophic nutrition). With this aim, N_2_ fixation activity and *nifH* diversity were explored in parallel with high-resolution dissolved organic matter (DOM) analysis along two transects in the Solomon and Bismarck Seas in the Southwest Pacific. This study area was chosen as a key site where the waters of South Pacific Gyre are channeled towards the Equator via low-latitude western boundary currents to feed the Pacific Equatorial Undercurrent [27]. The extremely high N_2_ fixation rates measured in the surface waters of the Solomon Sea (up to 610 nmol N L^-1^ d^-1^; [28]; Bonnet et al. unpublished) suggest that this area plays an important role in redistributing nutrients over the Equator [29], while the magnitude and controls of aphotic N_2_ fixation in these waters has not been explored before. We find that N_2_ fixation activity is correlated to several organic matter parameters, suggesting that mesopelagic N_2_ fixation is sustained by chemoorganoheterotrophic nutrition, providing new insights into the ecology of non-cyanobacterial diazotrophs in the ocean.

## Materials and Methods

**Fig 1:**
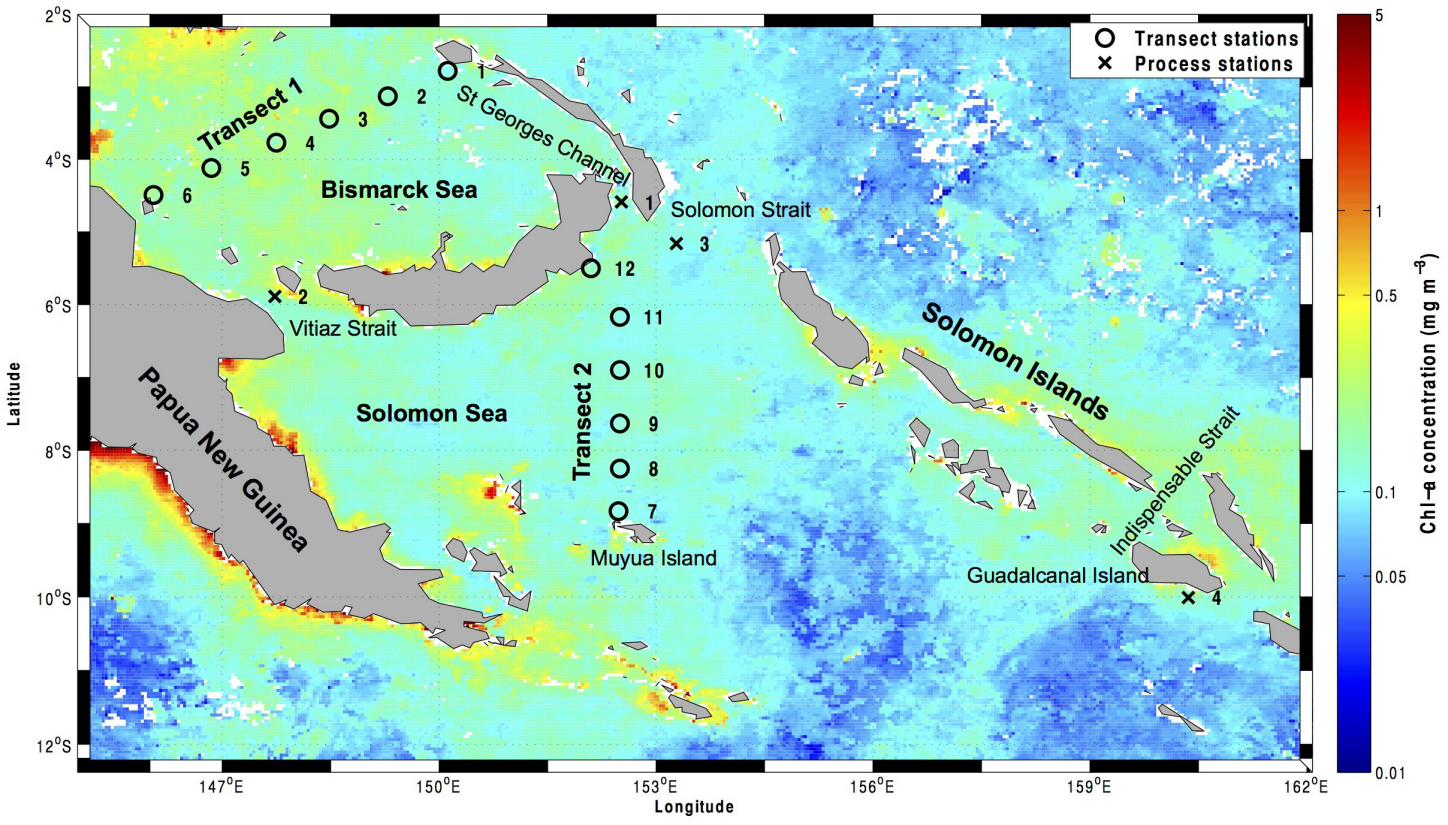
Stations sampled during the MoorSPICE cruise superimposed on a satellite image of chlorophyll *a* (Chl-a) concentrations. Chl-a data were obtained from the National Aeronautics and Space Administration (NASA) Goddard Earth Sciences Data and Information Services Center Giovanni (NASA GES DISC) online database for the month of March 2014. Transect stations are numbered from 1 to 12 and depicted by an open circle. Process stations (where DOM enrichment experiments were made) are numbered from 1 to 4 and depicted by a cross.

The MoorSPICE cruise took place from 1 to 31 March 2014 onboard the R/V *Thomas G*. *Thompson*. Seawater samples were taken along two transects: Transect 1 (stations 1–6, Bismarck Sea) and Transect 2 (stations 7–12, Solomon Sea) (Fig 1), at four depths in the mesopelagic zone (200 to 1000 m). All samples analyzed during this study were collected in international waters and did not require specific permissions. This study did not involve protected or endangered species. The sampling depths were chosen based on oxygen profiles observed real-time during CTD (conductivity temperature depth) casts onboard, with the aim of capturing zones of contrasted conditions or ‘ecotones’ where an increase in biological activity and diversity could be expected. Profiles of temperature, pressure and salinity were recorded using a SBE11+ CTD (Sea-Bird Electronics) equipped with oxygen and transmission sensors. All sensors were mounted on a rosette frame equipped with 24 Niskin bottles of 10 L.

### Inorganic nutrients

Samples for the determination of nitrate plus nitrite (NO_x_) and phosphate (PO_4_^3-^) concentrations were taken on acid-washed 20 mL polyethylene tubes, poisoned with 1% HgCl_2_, and stored at 4°C until analysis. The samples were analyzed on a AA3 Bran+Luebbe autoanalyzer with detection limits of 50 nM and 10 nM for NO_x_ and PO_4_^3-^, respectively.

### TEP

Duplicate water samples (400 mL each) were filtered onto 25 mm diameter 0.4 μm pore size polycarbonate filters and stained for 2 s with a 0.02% aqueous solution of Alcian blue in 0.06% acetic acid. The filters were stored at -20°C until analysis. For analysis the filters were soaked in 5 mL of 80% sulfuric acid and the absorbance was measured at 787 nm in 1 cm disposable polystyrene cuvettes using a DU 600 spectrophotometer (Beckman Coulter), and using ultrapure water as blanks. Three blanks were performed in each batch of samples every day (including staining and freezing in parallel to the samples). Each solution of Alcian blue was calibrated using a fresh standard solution of gum xanthan (GX) with a concentration of 25 mg L^-1^. The coefficient of variation of the replicates was ~17%. TEP concentrations (μg of gum xanthan equivalents per liter -μg GX Equiv L^-1^-) were measured according to Passow and Alldredge [30].

### DOM analysis by Fourier transform ion cyclotron resonance mass spectrometry (FT-ICR-MS)

DOM molecular composition was analyzed by ultrahigh-resolution mass spectrometry via the FT-ICR-MS technique. For this analysis, 2 L of seawater were acidified with hydrochloric acid to pH 2. DOM was then solid-phase extracted with 1 g modified styrene divinyl benzene polymer cartridges (PPL, Agilent) after Dittmar et al. [31]. After extraction, the cartridges were rinsed with acidified ultrapure water to remove remaining salts, dried by flushing with argon and eluted with 6 mL of methanol (HPLC-grade, Sigma-Aldrich). Extracts were stored in amber vials at -20°C. The extraction efficiency was on average 64% on a carbon basis. The concentration of extractable dissolved organic carbon (DOC) was determined in the solid-phase extracts after complete removal of the methanol and dissolution in ultrapure water.

The mass spectra were obtained at the University of Oldenburg on a 15 Tesla Solarix FT-ICR-MS (Bruker Daltonics) equipped with an electrospray ionization source applied in negative mode. DOM extracts were diluted to a final DOC concentration of 20 mg C L^-1^ in a 1:1 (v/v) mixture of ultrapure water and methanol. A total of 500 scans were accumulated per run in a mass window from 150 to 2000 Da. The spectra were mass-calibrated with an internal calibration list using the Bruker Daltonics Data Analysis software package. The detection limit and reproducibility of FT-ICR-MS analysis is described in Riedel and Dittmar [32]. The mass to charge, resolution and intensity were then exported and processed using in-house Matlab routines. All samples were measured in series in arbitrary order. Molecular formulae were assigned to masses with a minimum signal-to-noise ratio of 4 following the rules published in Koch et al. [33]. Signal intensities were normalized so that the sum of all signals was 1 for each given sample. To visualize compositional differences among samples, we performed a principal coordinate analysis (PCoA) on Bray-Curtis distance matrices, including all detected molecular formulae and their respective peak intensities. The PCoA scores were correlated against all hydrographic and biological variables measured in our study, as well as to the intensity of all the compounds detected in each sample.

### Bacterial abundance

Bacteria were enumerated using a FACSCalibur flow cytometer (BD Biosciences). The samples were fixed with 2% paraformaldehyde (final concentration) and stored at -80°C until analysis. Immediately before analysis, the thawed samples were stained with SYBR Green I (Invitrogen) at room temperature in the dark for 15 min. Fluorescent microspheres (Trucount and 2 μm beads; BD Biosciences) were added to the samples as an internal standard. The samples were run in medium mode for 1 min. Red (FL3) versus green (FL1) fluorescence, and green versus side scatter (SSC) cytograms were used to gate the bacteria using the FlowJo software.

### N_2_ fixation rates

N_2_ fixation rates were measured using the dissolved ^15^N_2_ method as described in Großkopf et al. [7], using 98% ^15^N_2_ from Euriso-top (subsidiary of Cambridge Isotope Laboratories). The ^15^N at% enrichment of ^15^N_2_-enriched seawater and samples labeled with ^15^N_2_ was measured using a Pfeiffer Vacuum Membrane inlet mass spectrometer (MIMS) according to Kana et al. [34]. The enrichment of our ^15^N_2_-enriched seawater varied from ~63 to 76%.

The samples to measure N_2_ fixation rates were collected in darkened triplicate 4.3 L polycarbonate bottles (Nalgene) fitted with septum screwcaps. ^15^N_2_-enriched seawater was added to 5% volume (i.e. 220 mL), enabling an initial ^15^N atom % enrichment of ~4%. The samples were incubated for 24 h in the dark in a temperature-controlled incubator at 8°C. After incubation, the samples were filtered through precombusted GF/F filters (Whatman), and the filters were stored at -20°C until analysis. Background (time zero δ^15^N) samples were taken at every sampling depth (four depths from 250 to 1000 m) at every other station. The samples were analyzed by continuous-flow isotope ratio mass spectrometry (IRMS) using an Integra2 Analyser (Integra CN). IRMS data were screened for significant δ^15^N changes (S1 Methods).

Commercial ^15^N_2_ gas stocks have recently been reported to be contaminated with variable amounts of ^15^N-labeled ammonium, nitrate, nitrite and nitrous oxide [35]. This is of special concern for sites of low diazotrophic activity, where N_2_ fixation rates could be masked by the uptake of other compounds. The contamination of the ^15^N_2_ gas stock used was measured and the potential overestimation of N_2_ fixation rates was calculated (S1 Methods).

### DOM addition experiments

DOM addition experiments were conducted in four selected ‘Process’ stations (depicted with a cross in Fig 1) to test whether the addition of sugars or amino acids enhanced N_2_ fixation activity. At these stations, water was sampled from a single depth located between 300 and 400 m, which was chosen to coincide with the dissolved oxygen minimum of the Subtropical Mode Water mass, the goal being to sample the same water mass at different ‘Process’ stations. The temperature, oxygen and salinity values at the time of sampling on these stations are shown in S1 Table.

At each station, seawater samples were collected into twelve acid-washed 4.3 L transparent polycarbonate bottles. Three bottles were used as time zero, and the contents of these bottles were filtered immediately after collection. One set of triplicates was amended with a mix of carbohydrate and small organic acids (39% glucose, 29% sodium acetate, and 32% sodium pyruvate (molar percentages), final total molar concentration of 1 μM), and another set of three bottles was amended with a mix of amino acids (20% leucine, 23% glutamic acid, and 56% alanine, to reach a final molar concentration of 1 μM). A final set of bottles was unamended and used as a control. After 24 h, ^15^N_2_ was added to all bottles that were then incubated for another 24 h to measure the response of N_2_ fixation rates to the addition of organic compounds.

### DNA extraction, clone library construction, primer design and qPCR

To sample DNA, 4.3 L of seawater was filtered through 0.2 μm Supor filters (Pall Corporation) using a peristaltic pump. The filters were shipped in dry ice and stored at -80°C until analysis. DNA was extracted using the Qiagen DNeasy Plant Mini Kit, as modified by Moisander et al. [36]. A nested PCR approach with degenerate *nifH* primers 1, 2, 3, and 4 [37] was used to create a *nifH* clone library. The first round of the nested PCR consisted of 25 μL reactions containing 2.5 μL 10x PCR buffer, 1.25 μL 50 mM MgCl_2_, 0.5 μL of 10 mM dNTPs, 1 μL primers nifH3 and nifH4 each (at 25 μM stock concentration) [37] (Eurofins MGM Operon), 0.11 μL Platinum Taq DNA polymerase (Life Technologies, Invitrogen), and 5 μL DNA template, adjusted to 25 μL with nuclease-free water. The volume of nuclease-free water was increased on the second round to include only 1 μL of template from the first round reaction, and primers replaced with niH1 and nifH2 [37]. PCR conditions for both the first and second round of the nested PCR were as follows: initial 95°C for 3 min, then for 31 cycles at 95°C for 30 s, 57°C for 30 s, and 72°C for 1 min, and final 72°C for 7 min.

PCR products were separated on a 1.2% TAE (Tris base, acetic acid and EDTA) gel, and bands were excised and purified using a GeneJET Gel Extraction Kit (Thermo Scientific). The products were cloned using the pGEM-T vector system (Promega). Plasmid DNA was purified using the Millipore Montage 96-well system (Billerica) and sequencing was done at the Massachusetts General Hospital sequencing facility (Cambridge, MA, USA). The sequences were trimmed in CLC Main Workbench 7, then imported into ARB [38]. Sequences were aligned to the previously HMMR aligned *nifH* database [39] and a neighbor-joining phylogenetic tree was built with selected sequences from the NCBI database. The tree was bootstrapped with 1000 repetitions using MEGA6-06.

Sequences close to Deltaproteobacteria, Gammaproteobacteria and Cluster III were chosen to design TaqMan (Life Technologies, Applied Biosystems) quantitative PCR (qPCR) primer-probe sets using Primer Express software (Life Technologies) (S1 Methods, S2 Table). Sequence data were deposited in the GenBank database under accession numbers KT025938 to KT026034.

## Results

The distribution of temperature, salinity, inorganic nutrients (NO_x_ and PO_4_^3-^) and total bacteria (cells mL^-1^) are described in the supporting information (S1 Results; S1 and S2 Figs).

### TEP

**Fig 2:**
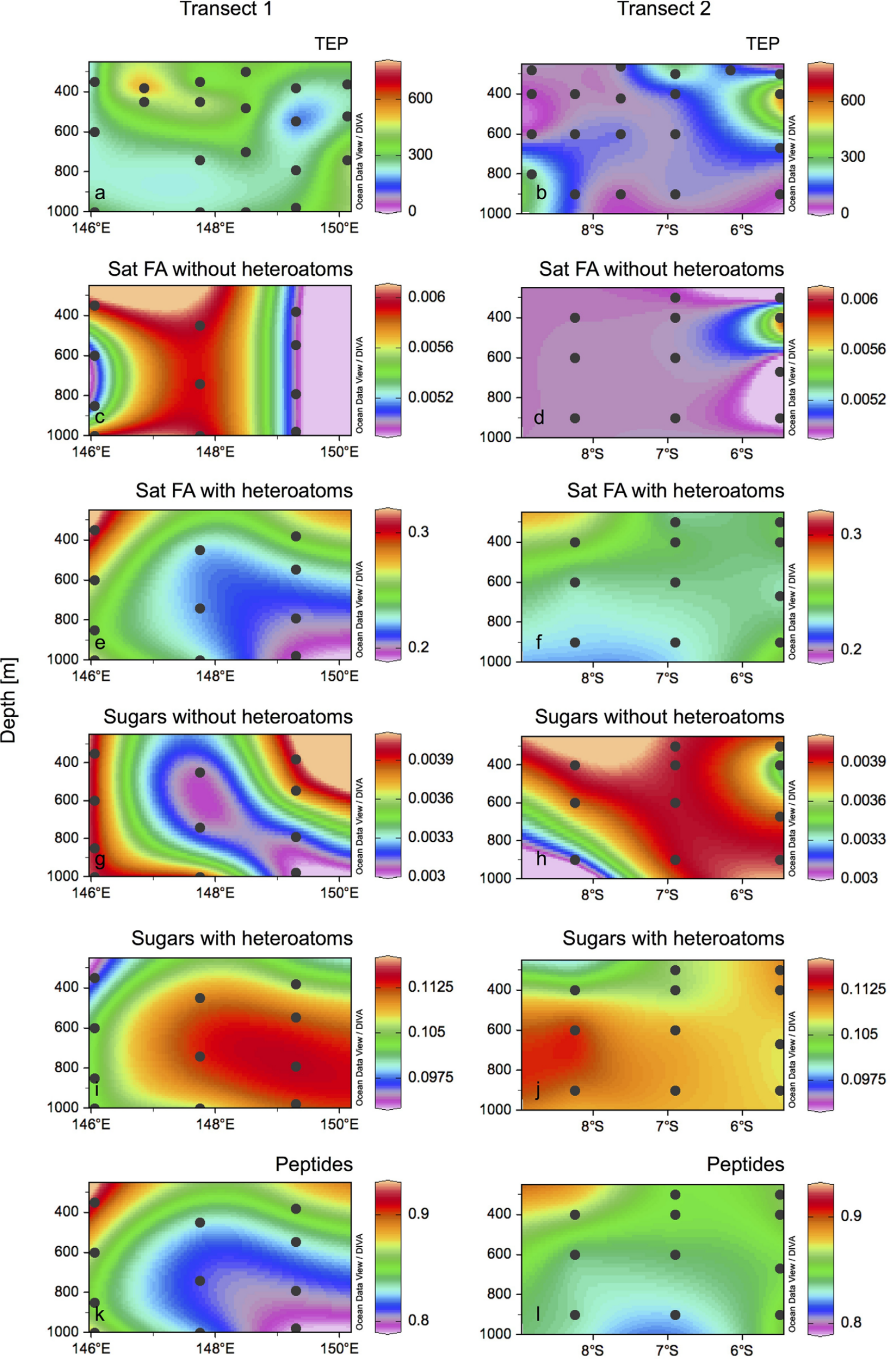
Distribution of organic matter compounds along transects 1 and 2. Transect 1 and Transect 2 (a, b) concentrations of TEP (μg GX Equiv L^-1^), (c, d) sum of relative peak intensities of saturated fatty acids without heteroatoms, (e, f) saturated fatty acids with heteroatoms, (g, h) sugars without heteroatoms, (i, j) sugars with heteroatoms, and (k, l) peptides.

TEP concentrations were higher in Transect 1 than in Transect 2 (*t*-student, p<0.05), showing a relatively patchy distribution (Fig 2A and 2B). Along Transect 1, TEP concentrations ranged between 250 and 400 μg GX Equiv L^-1^, with only some higher concentration patches towards the western part of the transect, and a lower concentration towards its eastern edge (Fig 2A). In Transect 2, the concentration of TEP was mostly <100 μg GX Equiv L^-1^ (but ranging from 40.8 to 536.6 μg GX Equiv L^-1^), with higher concentrations in the southern and northern ends of the transect (100–600 μg GX Equiv L^-1^; Fig 2B). Higher concentrations of TEP coincided with stations closer to the coast (Papua New Guinea coast on Transect 1, and Muyua and New Britain Islands on Transect 2).

### High-resolution analysis of DOM (FT-ICR-MS) and correlations with other variables

**Fig 3:**
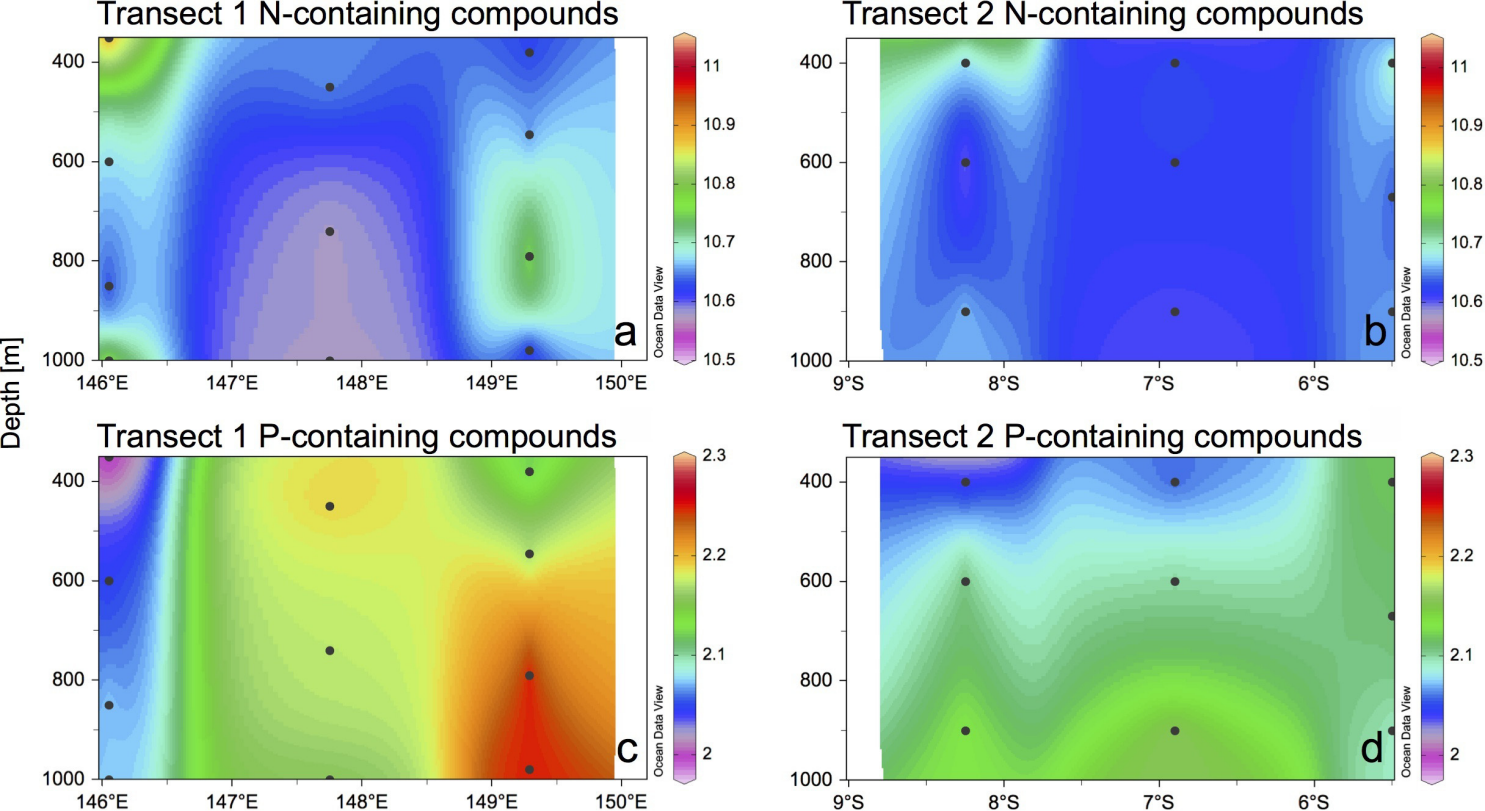
Sum of relative peak intensities of N-containing organic compounds. (a) Transect 1, and (b) Transect 2, and P-containing organic compounds in (c) Transect 1, and (d) Transect 2.

The high-resolution analysis of DOM resulted in the detection of ~3000 molecular formulae in each sample, covering a mass range of 155–1026 Da. Each molecular formula was assigned to a given compound group as described in Săntl-Temkiv et al. [40]. Because many structural alternatives may exist for a given molecular formula, these structural assignments are not necessarily unambiguous, but they provide a valuable overview of the otherwise very complex data. All compound groups presented and discussed in the following refer to the detected molecular formulae. 36–40% of all compounds detected were oxygen-poor (O/C<0.5) unsaturated aliphatics, and 20–22% were oxygen-rich (O/C>0.5) unsaturated aliphatics. Less than 8% of all compounds detected were polyphenols, while saturated fatty acids, sugars and peptides represented <4% of the total. The spatial distribution of compounds usually regarded as labile (i.e. molecular formulae of saturated fatty acids, sugars and peptides) was investigated (Fig 2C–2L). Transect 1 harbored more molecular formulae of saturated fatty acids with and without heteroatoms, sugars with and without heteroatoms, and peptides than Transect 2 (11 *versus* 16, 401 *versus* 576, 11 *versus* 16, 176 *versus* 357 and 1407 *versus* 1908 formulae, respectively). Adding up the intensities of all DOM compounds containing N or P revealed that Transect 1 harbored more DOM compounds with these heteroatoms than Transect 2 (15058 N-containing formulae in Transect 1 *versus* 14029 in Transect 2, and 2222 P-containing formulae in Transect 1 *versus* 2056 in Transect 2; Fig 3), thus co-varying with TEP concentrations (Fig 2A and 2B). This was especially prominent for P-containing compounds, which abounded in the eastern part of Transect 1 (Fig 3C). Of all formulae detected, those including N represented 33–37% and those containing P represented 5–6% of the total.

**Fig 4:**
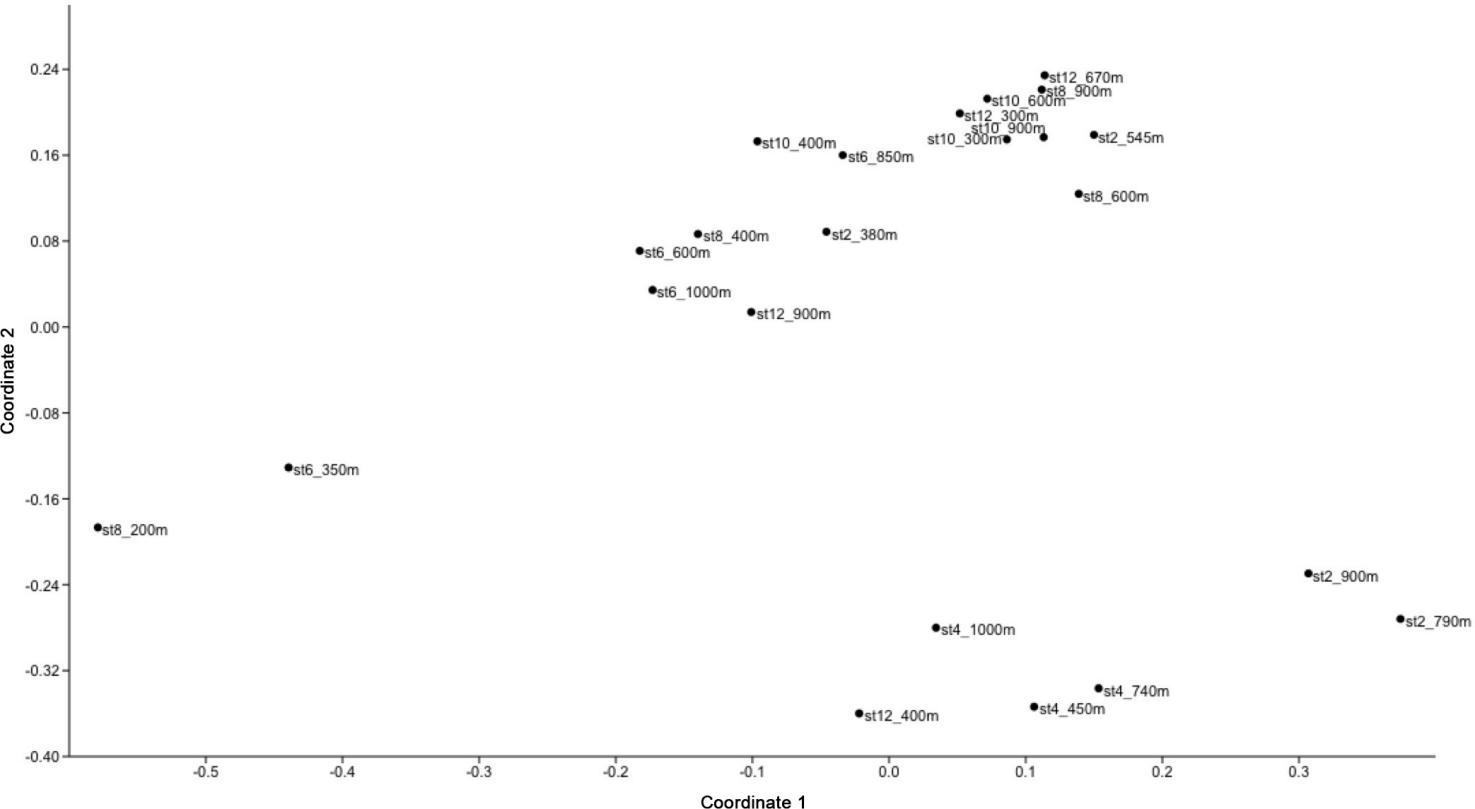
Multivariate ordinations of DOM compound relative peak intensities for 23 samples. Each point is labeled with the station and depth where the sample was collected (for the geographical location of the stations see Fig 1).

A principal coordinate analyses (PCoA) taking into account the relative signal intensities of all the DOM compounds of our samples indicated that the composition of Transect 1 samples was distinct from Transect 2 (Fig 4). The first coordinate explained 71% of the variation in the DOM composition, while the second coordinate explained 8%. Stations 2 and 4 (Transect 1) clustered together, while the rest of stations aggregated in another cluster. The samples corresponding to station 6 at 350 m and station 8 at 200 m appeared largely separated from the rest (Fig 4). The correlation of PCoA scores with all variables of interest (hydrographic parameters, inorganic nutrients, TEP, POC, N- and P-content of DOM, saturated fatty acids with/without heteroatoms, sugars with/without heteroatoms, peptides, bacterial abundance and N_2_ fixation rates; S4 Table) revealed that the first coordinate was negatively correlated with temperature and salinity, and hence positively correlated with inorganic nutrients. The first coordinate was also negatively correlated with the abundance of bacteria. The second coordinate was positively correlated with TEP and N_2_ fixation (note that these two variables are significantly correlated among themselves, see below).

The correlation coefficient of the first and second coordinate PCoA scores with the intensity of all DOM compounds detected in each sample (S3A Fig) revealed that, aside the molecular differences described above, there was a clear gradient in oxygen content of the molecular formulae among the two transects. In S3A Fig, the positive correlation coefficients (red) represent the more oxygenated formulae detected in Transect 1, while the negative correlation coefficients (blue) represent those less oxygenated formulae detected in Transect 2. The second PCoA component (S3B Fig) did not show clear patterns or any other consistent molecular trends.

### N_2_ fixation rates

N_2_ fixation rates ranged from undetectable (our detection limit was 0.062 nmol N L^-1^ d^-1^) to ~1 nmol N L^-1^ d^-1^. However, given the uncertainty of δ^15^N values at low PN masses, the δ^15^N values presented here have a variability of ±1.67‰ (S1 Methods). This variability could alter the value of our N_2_ fixation rates by ±8.34%. Overall, the uncertainty at low PN mass values and the minor contamination of the ^15^N_2_ indicate that the N_2_ fixation rates presented here are potentially variable by <10%.

**Fig 5:**
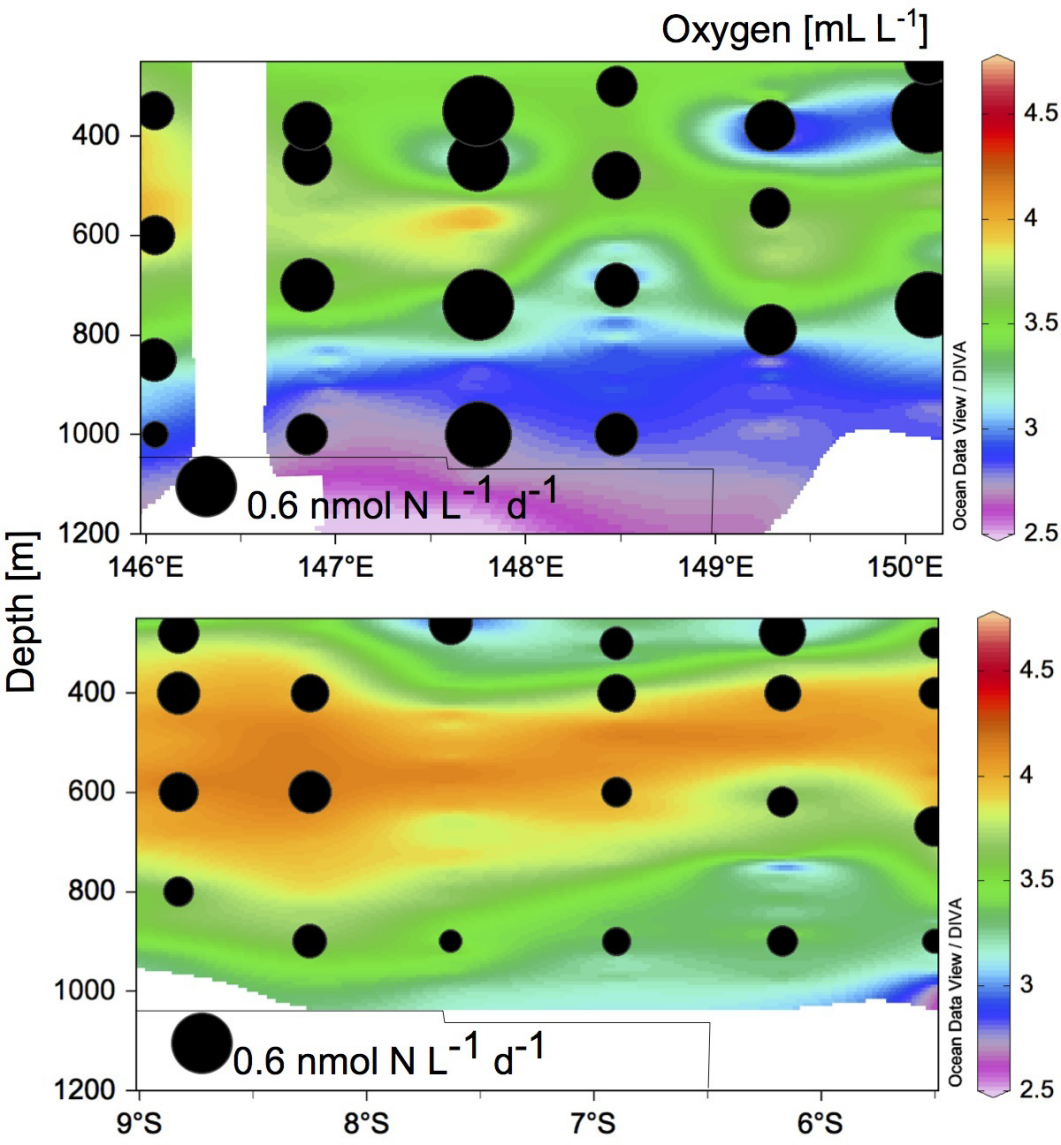
N_2_ fixation rates superimposed on oxygen concentrations. (a) N_2_ fixation rates on Transect 1, and (b) Transect 2. Oxygen concentrations refer to the color scale (mL L^-1^). The lower black circle indicates the reference size corresponding to 0.6 nmol N L^-1^ d^-1^.

N_2_ fixation rates were greater on Transect 1 (from undetectable to 0.9 nmol N L^-1^ d^-1^; Fig 5A) than in Transect 2 (from undetectable to 0.4 nmol N L^-1^ d^-1^; Fig 5B) (*t*-test, p = 0.001) coinciding with the lower oxygen concentrations on Transect 1 (2.5–3.5 mL L^-1^; Fig 5A) as compared to Transect 2 (3.5–4.2 mL L^-1^; Fig 5B). N_2_ fixation rates had a moderate significant negative correlation with oxygen concentrations (Pearson *r* = 0.382, p = 0.004), and positive moderate correlation with TEP concentration (Pearson *r* = -0.428, p = 0.02; S4 Table).

**Fig 6:**
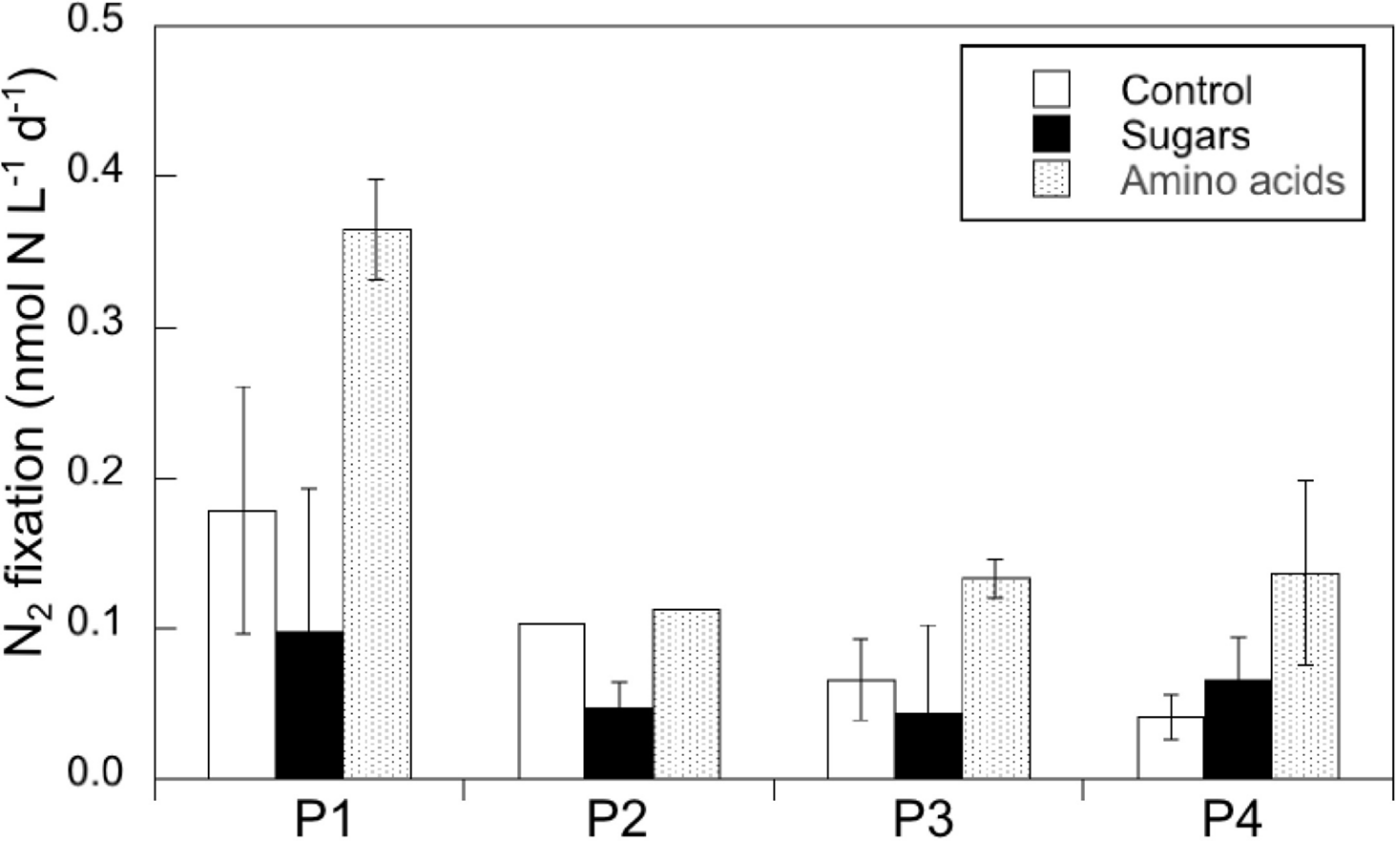
Response of N_2_ fixation rates to sugars and amino acids additions at Process stations 1–4. Error bars represent the standard deviation of the mean of the triplicates.

At Process stations, the addition of amino acids enhanced N_2_ fixation rates one to two-fold (although not at Process station 2; Fig 6), while the addition of sugars resulted in rates lower than the control (with the expectation of Process station 4; Fig 6). However, taking into account all of the Process stations, the N_2_ fixation rates were not statistically different among treatments (one-way repeated measures ANOVA, p = 0.07).

### *nifH* gene diversity and abundance

**Fig 7:**
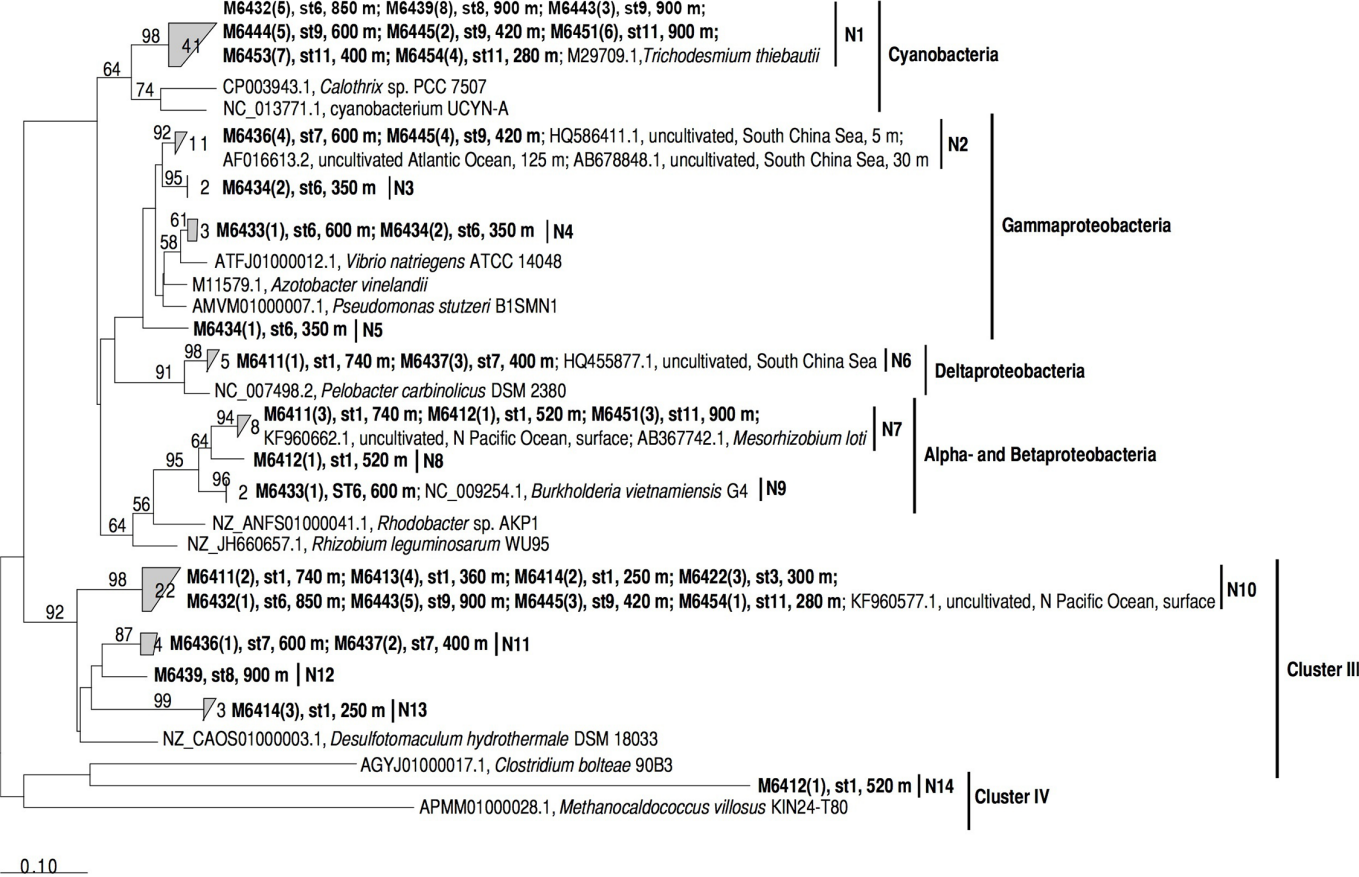
Neighbor-joining tree of partial *nifH* amino acid sequences. The sequences recovered from transects 1 and 2 are depicted in bold font. The number of clones recovered for each sample is indicated in parenthesis, and the station (st) and depth where the sample was recovered are shown. Clusters according to *nifH*-based phylogeny are shown to the right of the tree. Phylotypes are labeled as N1-N14 and cross-referenced with accession numbers in S2 Table (note that sequence M6432A05 is not included in the tree due to it being shorter). Reference amino acid sequence names are shown with GenBank accession, site, depth and clone numbers (where available). The sequences M6433A04 (N4), M6411A02 (N6) and M6413A02 (N10) were used for qPCR assays.

A *nifH* clone library was generated from DNA extracts obtained from both transects. Out of the 48 samples assayed for *nifH* 31 amplified, while negatives (no template controls) never showed bands. In total 96 sequences were included in a neighbor-joining phylogenetic tree together with cultivated and uncultivated representatives of different *nifH* clusters. The sequences from this study clustered with cyanobacteria, Alpha-, Beta-, Gamma-, and Deltaproteobacteria (Cluster I), Cluster III and Cluster IV, as defined by Zehr et al. [41] (Fig 7; S3 Table).

Out of all the sequences recovered, 42% were closely related with *Trichodesmium* sp., including a clone recovered from North Pacific Ocean, and to a cultured representative of *T*. *thiebautii*. These cyanobacterial sequences were mostly recovered from Transect 2 at various depths between 200 and 1000 m, but also from station 6 in Transect 1 at 850 m (Fig 7). The sequences from Transect 1 clustered with Alpha-, Beta-, Gamma-, and Deltaproteobacteria. Those close to Gammaproteobacteria represented 16% of the total, and were closely related to an uncultured bacterium from surface waters off the Cape Verde Islands (99% identity at the amino acid level, accession number AF016613.2). These Gammaproteobacteria-like sequences were recovered from 350 to 600 m at station 6 (Transect 1), and stations 7 and 9 (Transect 2). The Deltaproteobacteria-like sequences represented 4% of the total and were closely related to an uncultivated bacterium from the South China Sea (95% identity, accession number HQ455877.1). Sequences close to Alpha- and Betaproteobacteria were recovered from stations 1 and 7, and represented 8% of the clone library. These were related to an uncultivated bacterium from the North Pacific Ocean (99% identity, accession number KF960662.1), and to Rhizobiales such as *Mesorhizobium loti* (98% identity, accession number AB367742.1). One sequence recovered from station 6 at 600 m was closely related to *Burkholderia vietnamiensis*, which is close to other bacteria considered to be a contaminant associated with PCR reagents, but may also be present in natural environments [36]. The second most abundant group of sequences contained clustered with the *nifH* Cluster III (29%). These sequences were recovered from stations 1, 6, 7, 8, 9, 10 and 11, being the most widespread *nifH* cluster detected in this study. These sequences were related to uncultivated bacteria recovered from the North Pacific and the Arabian Sea, as well as to *Desulfotomaculum hydrothermale*, which is an anaerobic bacterium often found in hot springs. A single sequence clustered with Cluster IV at 360 m in station 1, which was moderately related to *Clostridium bolteae* (86%, Firmicutes, GenBank accession number AGYJ01000017.1), and shared 96% identity with a bacterium retrieved from the Eastern Tropical South Pacific OMZ (KF515795.1; [21]).

**Fig 8:**
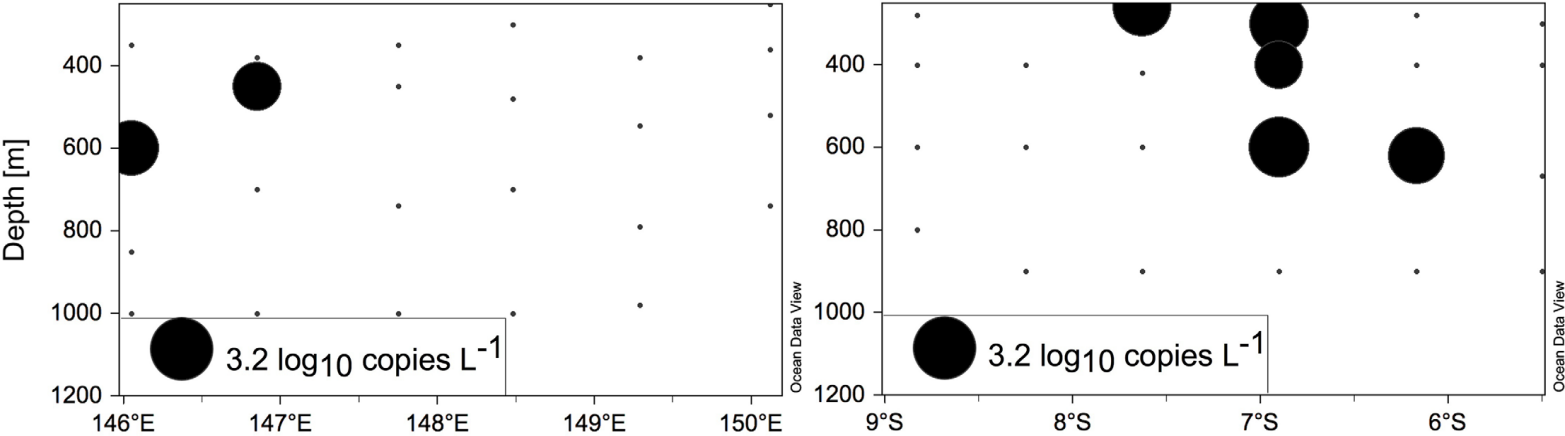
*NifH* copy abundance of the phylotype M6413A02 as determined by qPCR assays. The lower black circle represents the reference size corresponding to 3.2 log_10_ copies L^-1^ in (a) Transect 1, and (b) Transect 2.

The abundance of three selected phylotypes was assayed by qPCR using primer-probe sets designed in this study based on the clone library (S3 Table). The qPCR assays with a primer-probe set targeting a phylotype clustering near Deltaproteobacteria (M6411A02) resulted in detectable but not quantifiable amplification, while for the primer-probe set targeting a phylotype close to Gammaproteobacteria (M6433A04), amplification was below detection. The primer-probe set targeting a phylotype in Cluster III (M6413A02) amplified from samples obtained from some stations and depths, with abundances ranging from ~70 to ~900 *nifH* gene copies L^-1^ (or 0 to ~3 log_10_ *nifH* copies L^-1^; Fig 8). Only 10 out of the 48 samples assayed resulted in *nifH* gene copy abundance values above the detection and quantification limits (for primer-probe set M6413A02; Fig 8). On Transect 1, M6413A02 was detected at stations 5 and 6 with ~80 and ~300 *nifH* copies L^-1^. On Transect 2, this phylotype was detected at single depths at stations 9 (260 m) and 11 (620 m), and at three depths at station 10 (from 300 to 600 m). The detected abundances ranged from ~70 to ~900 *nifH* copies L^-1^.

## Discussion

Despite the fact that N_2_ fixation in deep waters is potentially conducted by heterotrophic prokaryotes and hence dependent on organic matter (chemoorganoheterotrophs), previous studies have only explored relationships between N_2_ fixation rates and TEP concentrations (e.g. [25]). This study builds up on previous aphotic N_2_ fixation studies (e.g. [21,25]), and for the first time explores the DOM pool in detail and relates it to N_2_ fixation activity and diversity in aphotic waters.

The N_2_ fixation rates measured here (undetectable to ~1 nmol N L^-1^ d^-1^) are in the range of those measured in other mesopelagic to bathypelagic zones of the Mediterranean Sea and Gulf of Aqaba (0.01–0.38 nmol N L^-1^ d^-1^; [25]), in the Eastern Tropical South Pacific (up to 1 nmol N L^-1^ d^-1^; [21]), or in the Southern California Bight (0.07 nmol N L^-1^ d^-1^; [26]). N_2_ fixation rates were higher on Transect 1 (from undetectable to 0.89 nmol N L^-1^ d^-1^) than on Transect 2 (undetectable to 0.35 nmol N L^-1^ d^-1^). These differences may be due to the differential hydrographic and biogeochemical context of the two transects. The waters of the Solomon Sea are fed by the South Equatorial Current, part of them flowing northwards through Vitiaz Strait, part of them flowing eastwards along the southern coast of New Britain (see Fig 1). The latter flow through St Georges Channel and eventually flow westwards along the Bismarck Sea [29]. Because these bifurcated currents wash the coasts of Papua New Guinea and New Britain, respectively, the waters at the exiting Vitiaz Strait and those over the Bismarck Sea are richer in trace metals than those in the core of the Solomon Sea, which come from the ultraoligotrophic South Pacific Gyre [42]. Trace metal levels and photic N_2_ fixation measurements suggest that the Bismarck Sea is more productive than the Solomon Sea [28,42], which supports the differences in organic matter load observed in the mesopelagic layer in this study (Fig 2). Indeed, Transect 1 had higher carbon fixation rates at the surface than Transect 2 (0.1–9.7 versus 0.2–1.8 μmol C L^-1^ d^-1^, respectively; Berthelot et al., unpublished), and also higher chlorophyll *a* concentrations (Fig 1). The sedimentation of organic matter produced at the surface could explain the higher TEP concentration (Fig 2A and 2B), higher relative peak intensity of presumably labile compounds such as saturated fatty acids, sugars and peptides (Fig 2C–2L), DOM compounds containing heteroatoms (N and P; Fig 3), and higher POC concentrations (up to 9.2 μM in Transect 1, and up to 5.2 μM in Transect 2, data not shown) of Transect 1 as compared to Transect 2. Nevertheless, the lateral transport of organic matter is likely important in this highly hydrodynamic area [27].

N_2_ fixation rates were moderately positively correlated with TEP, and negatively with oxygen and the peak intensity of peptides and sugars as determined by FT-ICR-MS (S4 Table). The negative correlation with oxygen may suggest an alleviation of nitrogenase enzyme destruction, rendering oxygen-poor zones as spots favorable for N_2_ fixation (e.g. [8]). However, the correlation found here is weak (Pearson *r* = -0.428), and the magnitude of N_2_ fixation rates is often similar at given stations where oxygen concentrations vary from ~2 to 4 mL L^-1^ in the mesopelagic water column (Fig 5A). Moreover, heterotrophic diazotrophs are abundant and widespread in fully oxygenated surface waters across the oceans [13] and active in aphotic waters of the Mediterranean Sea [25], suggesting that oxygen-poor conditions are not a prerequisite for aphotic N_2_ fixation, or alternatively that the diazotroph assemblages inhabiting OMZs is physiologically distinct form that of oxygenated aphotic waters. On the other hand, the positive correlation with TEP suggests that the presence of degradable organic matter represent favorable conditions for heterotrophic N_2_ fixation in the mesopelagic zone, as previously observed in the Mediterranean Sea and the Gulf of Aqaba [25]. The negative correlation of N_2_ fixation rates with peptides and sugars may however be the result of a complex regulation pattern. In a recent study Benzton-Tilia et al. [43] analyzed the genome of three strains isolated from the coast of Denmark. These authors found that while a high percentage of the genome was invested in the metabolism of relatively refractory compounds such as aromatic hydrocarbons, part of it was also devoted to the metabolism of labile compounds such as fatty acids and sugars. The metabolic profile of different heterotrophic diazotrophs may vary considerably among phylotypes and environments [14,20]. The high phylogenetic diversity of aphotic heterotrophic diazotrophs found in this and other studies (e.g. [22]) likely hides a variety of metabolic strategies, resulting in sometimes contradictory patterns (i.e. positive correlations with some labile compounds, but negative with others) when calculated for bulk community N_2_ fixation rates.

The correlation of the first principal coordinate with temperature, salinity and inorganic nutrients (S4 Table), likely reflects the influence of water mass distribution on the distribution of DOM compounds, which may also influence prokaryotic activity at depth [44]. Principal coordinate 1 as well as the peak intensities of N- and P-containing organic compounds were negatively correlated to bacterial abundance (all p<0.05; S4 Table), suggesting the impact of prokaryotic activity on *in situ* DOM pools. However, bacterial abundance was not correlated to the intensity of saturated fatty acids, sugars or peptides (S4 Table). An enhancement of N_2_ fixation rates was observed upon the addition organic compounds (although the experiments were performed in stations different to those of the transect, and the differences between treatments were not significantly different; Fig 6). The FT-ICR-MS approach used here does not detect masses below 150 Da, excluding small monomeric compounds and colloidal matter that contain potentially labile DOM fractions [31]. Therefore, the observed enhancement of N_2_ fixation rates upon the addition of the carbohydrates, small organic acids and amino acids used here (82 to 180 Da) is not directly comparable to the *in situ* DOM compounds detected with the FT-ICR-MS method. The addition of amino acids produced a higher N_2_ fixation response than the addition of sugars and acids (Fig 6). This pattern has been observed in other mesopelagic N_2_ fixation studies [21,25], and is likely explained by the preferential N and P cleavage as compared to carbon in prokaryotic enzymatic activities [45]. The nutritional requirements of marine heterotrophic diazotrophs and their potential variability among different phylotypes is in fact unknown.

Prokaryotic activity in the mesopelagic zone is thought to be supported by settling organic particles, DOM released *in situ* by migrating zooplankton, transported by overturning circulation water mass sinking processes, or produced *in situ* by chemolithoautotrophic bacteria [46]. Suspended or neutrally buoyant particles are believed to have a more homogeneous distribution in the water column and to sustain a larger prokaryotic activity than sinking particles [47]. Cluster III-like *nifH* sequences made up 30% of our clone library and were the most widespread among the two transects surveyed (Fig 7). The oxygen levels measured in these waters were well above those of hypoxic and suboxic zones in which Cluster III-like *nifH* sequences have been recovered before [24,26,48], however Cluster III sequences are often reported also from oxygenated epipelagic waters [49,50]. The distribution of Cluster III sequences here suggests that particles may have played an important role in sustaining the mesopelagic N_2_ fixation as they may provide oxygen-deficient loci where the nitrogenase enzyme would be protected from being destroyed by oxygen [20,51,52].

Of the observed heterotrophic diazotrophic phylotypes, it is unclear which ones contributed to the N_2_ fixation rates measured or in what proportion. Finding cyanobacterial sequences related to *Trichodesmium* at depths between 200 and 1000 m (Fig 7) was surprising, although similar observations have been made in the Sargasso Sea [22]. In the surface waters of the same transect, we observed *Trichodesmium* abundances at up to 10^5^ *nifH* copies L^-1^ (Berthelot et al., unpublished), which is in agreement with the maximum abundances observed in hotspots of *Trichodesmium* such as the Northwest Atlantic [11], and hence corroborates the importance of *Trichodesmium* in the sunlit layer of these waters. Indeed, several surface accumulations of *Trichodesmium* in the form of ‘slicks’ were visually observed at the surface in the Solomon Sea during this cruise. The presence of *Trichodesmium*-like *nifH* genes at mesopelagic depths suggests that the collapse of blooms can form aggregates that sink out of the euphotic zone. We discard the possibility that these *Trichodesmium*-like phylotypes were actively fixing N_2_ at these depths that were below the euphotic zone.

Heterotrophic diazotroph communities are usually highly diverse (e.g. [22,26]) making their quantification unfeasible, as the design and optimization of multiple qPCR primer-probe sets would be needed, yet the low abundances makes quantification challenging [53]. The high diversity of heterotrophic diazotroph communities suggests that the N_2_ fixation rates observed in this study could be accomplished by a variety of phylotypes at low abundances, rather than by a single phylotype present at abundances high *nifH* copies abundances. Nevertheless, the use of the dissolved ^15^N_2_ method and the negligible effect of ^15^N_2_ gas stock contamination observed strongly support the reliability of the N_2_ fixation rates and the importance of heterotrophic N_2_ fixation as significant flux in the ocean, even below the euphotic zone. Indeed, the mesopelagic N_2_ fixation rates presented here integrated between 200 and 1000 m represent 25% of the N_2_ fixed between 5 and 70 m in the overlying sunlit layer (Berthelot et al., unpublished), highlighting the importance of this flux. Single-cell approaches are needed to discern the fractional contribution of different heterotrophic diazotroph phylotypes to *in situ* N_2_ fixation rates.

The interactions between organic matter and microbes can only be revealed by integrative approaches where equal efforts are made on chemical compound characterization and functional and molecular microbial diversity [54]. To the best of our knowledge, the present study is the first to combine these disciplines to elucidate mesopelagic heterotrophic N_2_ fixation patterns in the ocean. Although our data cannot confirm that the heterotrophic diazotrophs detected actively take up the DOM compounds observed, the differential distribution of organic matter observed between the two transects surveyed, in parallel with distinct patterns of N_2_ fixation, suggests that these microorganisms benefit from *in situ* DOM pools, which provide them with energy to support their N_2_ fixation activity. These results are a step towards a better understanding of mesopelagic diazotrophy, a process which could potentially increase global N inputs to the ocean.

## Supporting Information

S1 Fig Profiles of hydrographic variables and nutrient concentrations. (a) temperature (°C) for **(A)** Transect 1, and **(B)** Transect 2, and the same order for each transect with variables **(C-D)** salinity, (Figures E-F) NO_x_ (nitrate + nitrite; μM), and **(G-H)** PO_4_^3-^ (μM). Measurement points are shown with dots.
(TIFF)

S2 Fig Profiles of bacterial abundance. **(A)** Total bacterial abundance (cells mL^-1^) in Transect 1, and **(B)** Transect 2.
(TIFF)

S3 Fig Van Krevelen diagrams of all molecular formulae detected in the samples. The color code is the correlation coefficient of **(A)** the first and **(B)** the second coordinate PCoA scores with the intensity of all molecular formulae.
(TIFF)

S1 Methods Details on IRMS and qPCR methods. (DOCX)

S1 Results Description of hydrographic data, inorganic nutrient concentrations, and bacterial counts. (DOCX)

S1 Table *In situ* parameters measured at process stations where DOM addition experiments were performed. (DOCX)

S2 Table qPCR primers and TaqMan probes designed for this study. (DOCX)

S3 Table List of clones and their corresponding GenBank accession number, phylotype and cluster. (DOCX)

S4 Table Pearson correlation coefficients among hydrographic, chemical and biological variables, as well as the first four coordinate scores derived from principal coordinate analysis (PCoA) of dissolved organic matter (DOM) compounds relative peak intensities. (DOCX)

## References

[1] Dugdale RC, Goering JJ. Uptake of new and regenerated forms of nitrogen in primary productivity. Limnol Oceanogr. 1967; 196–206.

[2] Capone DG, Burns JA, Montoya JP, Subramaniam A, Mahaffey C, Gunderson T, et al Nitrogen fixation by *Trichodesmium* spp.: An important source of new nitrogen to the tropical and subtropical North Atlantic Ocean. Global Biogeochem Cycles. 2005;19 10.1029/2004GB002331

[3] Mahaffey C, Michaels AF, Capone DG. The conundrum of marine N2 fixation. Am J Sci. 2005;305: 546–595. 10.2475/ajs.305.6-8.546

[4] Codispoti LA. An oceanic fixed nitrogen sink exceeding 400 Tg N a^-1^ vs the concept of homeostasis in the fixed-nitrogen inventory. Biogeosciences. 2007;4: 233–253. 10.5194/bg-4-233-2007

[5] Benavides M, Voss M. Five decades of N2 fixation research in the North Atlantic Ocean. FMARS 2015;2: 1–40. 10.3389/fmars.2015.00040

[6] Knapp AN. The sensitivity of marine N2 fixation to dissolved inorganic nitrogen. FMICB. 2012;3: 1–14. 23091472 10.3389/fmicb.2012.00374 PMC3476826

[7] Großkopf T, Mohr W, Baustian T, Schunck H, Gill D, Kuypers MMM, et al Doubling of marine dinitrogen-fixation rates based on direct measurements. Nature. 2012;488: 361–364. 10.1038/nature11338 22878720

[8] Loescher CR, Großkopf T, Desai FD, Gill D, Schunck H, Croot PL, et al Facets of diazotrophy in the oxygen minimum zone waters off Peru. ISME J; 2014;8: 2180–2192. 10.1038/ismej.2014.71 24813564 PMC4992080

[9] Capone DG, Zehr JP, Paerl HW, Bergman B, Carpenter EJ. *Trichodesmium*, a globally significant marine cyanobacterium. Science. 1997;276: 1221–1229. 10.1126/science.276.5316.1221

[10] Zehr JP, Mellon MT, Zani S. New nitrogen-fixing microorganisms detected in oligotrophic oceans by amplification of nitrogenase (*nifH*) genes. Appl Environ Microbiol; 1998;64: 3444–3450. 9726895 10.1128/aem.64.9.3444-3450.1998 PMC106745

[11] Luo YW, Doney SC, Anderson LA, Benavides M, Berman-Frank I, Bode A, et al Database of diazotrophs in global ocean: abundance, biomass and nitrogen fixation rates. Earth Syst Sci Data. 2012;4: 47–73. 10.5194/essd-4-47-2012

[12] Moisander PH, Beinart RA, Hewson I, White AE, Johnson KS, Carlson CA, et al Unicellular cyanobacterial distributions broaden the oceanic N2 fixation domain. Science. 2010;327: 1512–1514. 10.1126/science.1185468 20185682

[13] Farnelid H, Andersson AF, Bertilsson S, Al-Soud WA, Hansen LH, Sørensen S, et al Nitrogenase gene amplicons from global marine surface waters are dominated by genes of non-cyanobacteria. PLOS ONE. 2011;6: e19223 10.1371/journal.pone.0019223.t001 21559425 PMC3084785

[14] Farnelid H, Riemann L. Heterotrophic N2-fixing bacteria: overlooked in the marine nitrogen cycle? In: Couto GN, editor. Nitrogen Fixation Research Progress. New York: Nova Science Publishers, Inc; 2008 pp. 409–423.

[15] Farnelid H, Bentzon-Tilia M, Andersson AF, Bertilsson S, Jost G, Labrenz M, et al Active nitrogen-fixing heterotrophic bacteria at and below the chemocline of the central Baltic Sea. ISME J. 2013;7: 1413–1423. 10.1038/ismej.2013.26 23446833 PMC3695292

[16] Halm H, Lam P, Ferdelman TG, Lavik G, Dittmar T, LaRoche J, et al Heterotrophic organisms dominate nitrogen fixation in the South Pacific Gyre. ISME J. 2011;6: 1238–1249. 10.1038/ismej.2011.182 22170429 PMC3358028

[17] Moisander PH, Serros T, Paerl RW, Beinart RA, Zehr JP. Gammaproteobacterial diazotrophs and *nifH* gene expression in surface waters of the South Pacific Ocean. ISME J. 2014;8: 1962–1973. 10.1038/ismej.2014.49 24722632 PMC4184014

[18] Sohm JA, Webb EA, Capone DG. Emerging patterns of marine nitrogen fixation. Nat Rev Microbiol. 2011;9: 499–508. 10.1038/nrmicro2594 21677685

[19] Bentzon-Tilia M, Traving SJ, Mantikci M, Knudsen-Leerbeck H, Hansen JORL, Markager S, et al Significant N2 fixation by heterotrophs, photoheterotrophs and heterocystous cyanobacteria in two temperate estuaries. ISME J. 2014;9: 273–285. 10.1038/ismej.2014.119 25026373 PMC4303622

[20] Riemann L, Farnelid H, Steward GF. Nitrogenase genes in non-cyanobacterial plankton: prevalence, diversity and regulation in marine waters. Aquat Microb Ecol. 2010;61: 235–247. 10.3354/ame01431

[21] Bonnet S, Dekaezemacker J, Turk-Kubo KA, Moutin T, Hamersley RM, Grosso O, et al Aphotic N2 Fixation in the Eastern Tropical South Pacific Ocean. PLOS ONE. 2013;8: e81265 10.1371/journal.pone.0081265.s001 24349048 PMC3861260

[22] Hewson I, Moisander PH, Achilles KM, Carlson CA, Jenkins BD, Mondragon EA, et al Characteristics of diazotrophs in surface to abyssopelagic waters of the Sargasso Sea. Aquat Microb Ecol. 2007;46: 15–30. 10.3354/ame046015

[23] Deutsch C, Sarmiento JL, Sigman DM, Gruber N, Dunne JP. Spatial coupling of nitrogen inputs and losses in the ocean. Nature. 2007;445: 163–167. 10.1038/nature05392 17215838

[24] Fernández C, Farías L, Ulloa O. Nitrogen Fixation in Denitrified Marine Waters. PLOS ONE. 2011;6: e20539 10.1371/journal.pone.0020539.t001 21687726 PMC3110191

[25] Rahav E, Bar-Zeev E, Ohayon S, Elifantz H, Belkin N, Herut B, et al Dinitrogen fixation in aphotic oxygenated marine environments. FMICB. 2013;4: 1–11. 23986748 10.3389/fmicb.2013.00227 PMC3753716

[26] Hamersley MR, Turk KA, Leinweber A, Gruber N, Zehr JP, Gunderson T, et al Nitrogen fixation within the water column associated with two hypoxic basins in the Southern California Bight. Aquat Microb Ecol. 2011;63: 193–205. 10.3354/ame01494

[27] Grenier M, Cravatte S, Blanke B, Menkes C, Koch-Larrouy A, Durand F, et al From the western boundary currents to the Pacific Equatorial Undercurrent: modeled pathways and water mass evolutions. J Geophys Res. 2011;116: C12044 10.1029/2011JC007477

[28] Bonnet S, Biegala IC, Dutrieux P, Slemons LO, Capone DG. Nitrogen fixation in the western equatorial Pacific: Rates, diazotrophic cyanobacterial size class distribution, and biogeochemical significance. Global Biogeochem Cycles. 2009;23: GB3012 10.1029/2008GB003439

[29] Ganachaud A, Cravatte S, Melet A, Schiller A, Holbrook NJ, Sloyan BM, et al The Southwest Pacific Ocean circulation and climate experiment (SPICE). J Geophys Res Oceans. 2014;119: 7660–7686. 10.1002/2013JC009678

[30] Passow U, Alldredge AL. A dye-binding assay for the spectrophotometric measurement of transparent exopolymer particles (TEP). Limnol Oceanogr. 1995;40: 1326–1335. 10.4319/lo.1995.40.7.1326

[31] Dittmar T, Koch B, Hertkorn N, Kattner G. A simple and efficient method for the solid-phase extraction of dissolved organic matter (SPE-DOM) from seawater. Limnol Oceanogr Methods. 2008;6: 230–235. 10.4319/lom.2008.6.230

[32] Riedel T, Dittmar T. A Method Detection Limit for the Analysis of Natural Organic Matter via Fourier Transform Ion Cyclotron Resonance Mass Spectrometry. Anal Chem. 2014;86: 8376–8382. 10.1021/ac501946m 25068187

[33] Koch BP, Dittmar T, Witt M, Kattner G. Fundamentals of molecular formula assignment to ultrahigh resolution mass data of natural organic matter. Anal Chem. 2007;79: 1758–1763. 10.1021/ac061949s 17297983

[34] Kana TM, Darkangelo C, Hunt MD, Oldham JB, Bennett GE, Cornwell JC. Membrane inlet mass spectrometer for rapid high-precision determination of N2, O2, and Ar in environmental water samples. Anal Chem. 1994;66: 4166–4170.

[35] Dabundo R, Lehmann MF, Treibergs L, Tobias CR, Altabet MA, Moisander PH, et al The Contamination of Commercial ^15^N2 Gas Stocks with ^15^N-Labeled Nitrate and Ammonium and Consequences for Nitrogen Fixation Measurements. PLOS ONE. 2014;9: e110335 10.1371/journal.pone.0110335 25329300 PMC4201487

[36] Moisander PH, Beinart RA, Voss M, Zehr JP. Diversity and abundance of diazotrophic microorganisms in the South China Sea during intermonsoon. ISME J. 2008;2: 954–967. 10.1038/ismej.2008.51 18528417

[37] Zehr JP, Turner PJ. Nitrogen fixation: Nitrogenase genes and gene expression Methods in microbiology. London: Academic Press, Ltd; 2001 pp. 271–286.

[38] Ludwig W, Strunk O, Westram R, Richter L, Meier H, Yadhukumar, et al ARB: a software environment for sequence data. Nucleic Acids Res. 2004;32: 1363–1371. 10.1093/nar/gkh293 14985472 PMC390282

[39] Heller P, Tripp HJ, Turk-Kubo K, Zehr JP. ARBitrator: a software pipeline for on-demand retrieval of auto-curated *nifH* sequences from GenBank. Bioinformatics. 2014;30: 2883–2890. 10.1093/bioinformatics/btu417/-/DC1 24990605

[40] Šantl-Temkiv T, Finster K, Dittmar T, Hansen BM, Thyrhaug R, Nielsen NW, et al Hailstones: a window into the microbial and chemical inventory of a storm cloud. PLOS ONE. 2013;8: e53550 10.1371/journal.pone.0053550.g005 23372660 PMC3553149

[41] Zehr JP, Jenkins BD, Short SM, Steward GF. Nitrogenase gene diversity and microbial community structure: a cross-system comparison. Environ Microbiol. 2003;5: 539–554. 10.1046/j.1462-2920.2003.00451.x 12823187

[42] Slemons LO, Murray JW, Resing J, Paul B, Dutrieux P. Western Pacific coastal sources of iron, manganese, and aluminum to the Equatorial Undercurrent. Global Biogeochem Cycles. 2010;24: GB3024 10.1029/2009GB003693

[43] Bentzon-Tilia M, Severin I, Hansen LH, Riemann L. Genomics and Ecophysiology of Heterotrophic Nitrogen-Fixing Bacteria Isolated from Estuarine Surface Water. mBio. 2015;6: e00929–15. 10.1128/mBio.00929-15 26152586 PMC4495170

[44] Reinthaler T, Álvarez-Salgado XA, Álvarez M, van Aken HM, Herndl GJ. Impact of water mass mixing on the biogeochemistry and microbiology of the Northeast Atlantic Deep Water. Global Biogeochem Cycles. 2013;27: 1151–1162. 10.1002/2013GB004634 24683294 PMC3966262

[45] Grossart H-P, Ploug H. Microbial degradation of organic carbon and nitrogen on diatom aggregates. Limnol Oceangr. 2001;46: 267–277.

[46] Arístegui J, Gasol JM, Duarte CM, Herndl GJ. Microbial oceanography of the dark ocean's pelagic realm. Limnol Oceanogr. 2009;54: 1501–1529. 10.4319/lo.2009.54.5.1501

[47] Baltar F, Arístegui J, Gasol JM, Sintes E, Herndl GJ. Evidence of prokaryotic metabolism on suspended particulate organic matter in the dark waters of the subtropical North Atlantic. Limnol Oceangr. 2009;54: 182–193. 10.4319/lo.2009.54.1.0182

[48] Jayakumar A, Al-Rshaidat MMD, Ward BB, Mulholland MR. Diversity, distribution, and expression of diazotroph *nifH* genes in oxygen-deficient waters of the Arabian Sea. FEMS Microbiol Ecol. 2012;82: 597–606. 10.1111/j.1574-6941.2012.01430.x 22697171

[49] Church MJ, Jenkins BD, Karl DM, Zehr JP. Vertical distributions of nitrogen-fixing phylotypes at Stn ALOHA in the oligotrophic North Pacific Ocean. Aquat Microb Ecol. 2005;38: 3–14. 10.3354/ame038003

[50] Langlois RJ, Hummer D, LaRoche J. Abundances and distributions of the dominant *nifH* phylotypes in the northern Atlantic Ocean. Appl Environ Microbiol. 2008;74: 1922–1931. 10.1128/AEM.01720-07 18245263 PMC2268318

[51] Paerl HW. Microzone formation: its role in the enhancement of aquatic N2 fixation. Limnol Oceanogr. 1985;30: 1246–1252. 10.4319/lo.1985.30.6.1246

[52] Paerl HW, Prufert LE. Oxygen-poor microzones as potential sites of microbial N2 fixation in nitrogen-depleted aerobic marine waters. Appl Environ Microbiol. Am Soc Microbiol; 1987;53: 1078–1087. 10.1128/aem.53.5.1078-1087.1987 PMC203813 16347337

[53] Turk-Kubo KA, Karamchandani M, Capone DG, Zehr JP. The paradox of marine heterotrophic nitrogen fixation: abundances of heterotrophic diazotrophs do not account for nitrogen fixation rates in the Eastern Tropical South Pacific. Environ Microbiol. 2014;16: 3095–3114. 10.1111/1462-2920.12346 24286454

[54] Kujawinski EB. The impact of microbial metabolism on marine dissolved organic matter. Annu Rev Marine Sci. 2011;3: 567–599. 10.1146/annurev-marine-120308-081003 21329217

