# Supplementary figures and images for: Mesopelagic N2 Fixation Related to Organic Matter Composition in the Solomon and Bismarck Seas (Southwest Pacific)
Source: PLoS One. 2015 Dec 11;10(12):e0143775. doi: 10.1371/journal.pone.0143775 (PMC4684240; doi:10.1371/journal.pone.0143775)

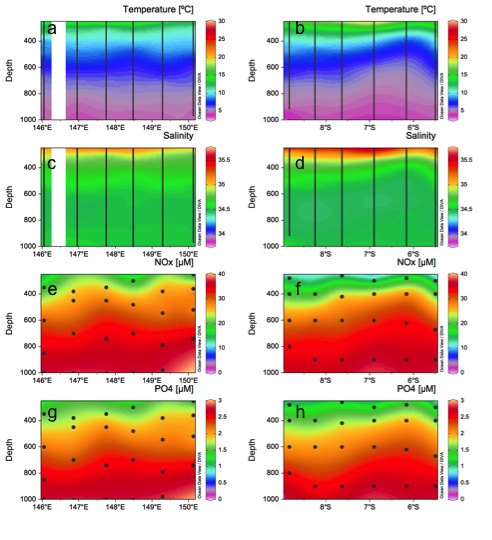

Supplement: S1 Fig — (a) temperature (°C) for (A) Transect 1, and (B) Transect 2, and the same order for each transect with variables (C-D) salinity, (Figures E-F) NOx (nitrate + nitrite; μM), and (G-H) PO4 3- (μM). Measurement points are shown with dots. (TIFF) [file pone.0143775.s001.tiff]

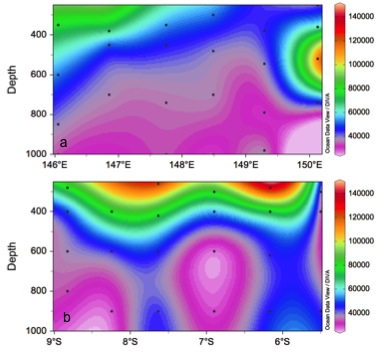

Supplement: S2 Fig — (A) Total bacterial abundance (cells mL-1) in Transect 1, and (B) Transect 2. (TIFF) [file pone.0143775.s002.tiff]

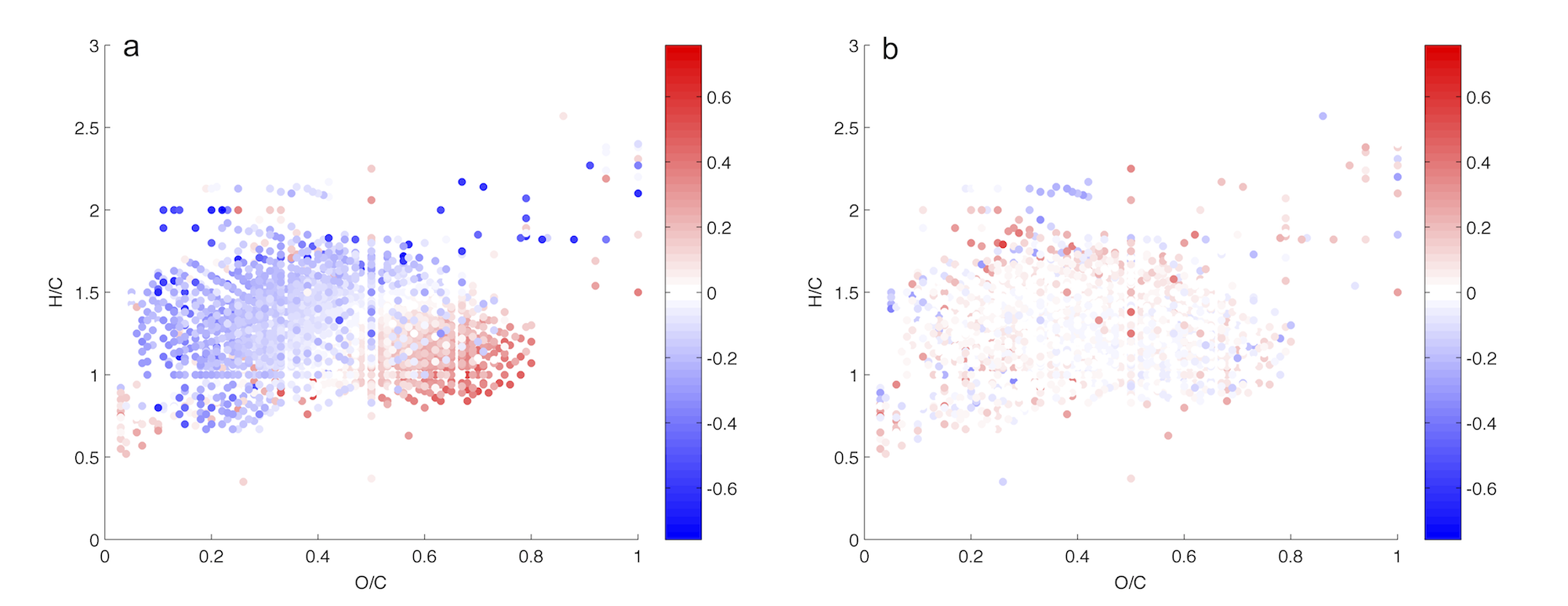

Supplement: S3 Fig — The color code is the correlation coefficient of (A) the first and (B) the second coordinate PCoA scores with the intensity of all molecular formulae. (TIFF) [file pone.0143775.s003.tiff]
