# Supplementary material for: Mesopelagic N2 Fixation Related to Organic Matter Composition in the Solomon and Bismarck Seas (Southwest Pacific)
Source: PLoS One. 2015 Dec 11;10(12):e0143775. doi: 10.1371/journal.pone.0143775 (PMC4684240; doi:10.1371/journal.pone.0143775)
Supplement: S1 Methods — (DOCX) [file pone.0143775.s004.docx]

**S1 Methods**

IRMS data screening and ^15^N_2_ gas contamination

Our ^15^N_2_ gas was checked for contamination as described in Dabundo *et al.* [1], and a model provided by these authors was used to estimate how much our rates may be overestimated due to ^15^N_2_ gas contamination (see Dabundo *et al.*, 2014). The contamination of the ^15^N_2_ gas stock used in this study was calculated to be 1.4 x 10^-8^ mol of ^15^NO_3_^-^ per mol of ^15^N_2_, and 1.1x10^-8^ mol ^15^NH_4_^+^ per mol of ^15^N_2_. Assuming these low contamination levels to our measurements using the model described in Dabundo *et al.* [1] amended by the authors with a modification to also include N_2_ gas dissolution variability with temperature and salinity as described in Weiss [2] (Dabundo and Granger, personal communication), indicates that the N_2_ fixation rates in this study could be overestimated by 0.85 ± 0.59%.

To remove the influence of background noise on the N_2_ fixation rates, all data below the linearity limit of the IRMS (2.32 µg N) were first removed. Particulate nitrogen (PN) masses ranged from 1.8 to 15.3 µg N, thus only 3 samples out of 179 were below the linearity limit. Since low PN masses give variable δ^15^N values, in a next step of data screening all δ^15^N values that were three times greater than the standard deviation of IAEA standards in the same mass and δ^15^N range than the samples were selected. The standard deviation among these measurements was δ^15^N = 1.67‰. This variability was taken into account to calculate the potential variability of N_2_ fixation rates. Finally, the remaining δ^15^N values that were significantly different from their corresponding time zero δ^15^N values (*t*-test, p=0.004) were kept. After this data screening procedure, 134 δ^15^N values out of the original 179 measurements made were retained (i.e. ~74% of the original data were used).

Considering the PN linearity limit (2.32 µg N), and three times the standard deviation of our background (time zero δ^15^N) values (4.22‰), our usual filtration volume (4.3 L) and incubation time (24 h), we set our volumetric N_2_ fixation rate detection limit at 0.062 nmol N L^-1^ d^-1^.

qPCR primer-probe design and reaction conditions

To prepare qPCR standards, plasmids were linearized using NsiI enzyme (New England Biolabs), the success of the linearization checked on a gel, and the rest of the reactions purified using a GeneJET PCR purification kit (Thermo Scientific, Waltham, MA, USA). Concentrations of linearized plasmid were determined using the Picogreen assay and a SpectraMax M2 plate reader (Molecular Devices, Sunnyvale, CA, USA). A dilution series of the plasmids was created representing 10^0^- 10^7^ *nifH* gene copies per reaction. The efficiency of standard curves varied from 93.96 to 100.5%. The standard series was run in parallel with each of the samples. Standard curves were built by linear regression of threshold cycle (C_t_) and log_10_ gene copies per reaction using duplicate standards ranging from 10^7^ to 10^1^ gene copies.

Cross reactivity of the designed primer-probe sets was checked *in silico* by comparing them against the *nifH* database from Dr. Jonathan Zehr at the University of California Santa Cruz (http://pmc.ucsc.edu/~wwwzehr/research/database/) and the NCBI database using BLASTn. Cross reactivity of the designed primer-probe sets was also tested empirically, using the dilution series of each of the linearized plasmid minipreps, and previously designed diazotroph primer-probe sets targeting UCYN-A, *Trichodesmium* [3], Het-1 [4], UCYN-B (*Crocosphaera watsonii*) [5] and γ-24774A11 [5,6]. None of the non-target plasmid standards amplified with the new qPCR primer probe sets.

The qPCR reactions consisted of 10 µL ABI TaqMan Gene Expression Master Mix (Life Technologies, Applied Biosystems, Foster City, CA, USA), 6.4 µL nuclease-free water, 0.4 µM and 0.2 µM final concentrations of primers and probe, respectively, and 1.6 µL DNA template. All samples and standards were analyzed in duplicate. The reactions were run on a StepOnePlus Real-Time PCR system (Life Technologies). Duplicate no template controls (NTC) were included in all runs. Inhibition tests were carried out for all samples by adding 1.6 µL of both the sample and the 10^5^ standard to each sample well. The efficiencies of inhibition test runs ranged from 97.3 - 101.73%, thus significant inhibition of amplification was not present in the samples. The limit of detection (LOD) and detected but not quantifiable (DNQ) limits were considered as 1 and 8 gene copies per reaction, respectively (data not shown). Cases where amplification was below LOD were designated a value of 0 in the data set, whereas samples that amplified above LOD but below DNQ were designated a conservative value of 1 *nifH* copy per L^-1^. The sequences of the primer-probe sets designed in this study are shown in S2 Table.

The M6413A02 probe should detect all clones from this study within the phylotype N10; these all had 0-1 mismatches to the three oligonucleotides combined. The best match we found to this phylotype that was from another study (KF960577.1) had 1-2 mismatches in each oligonucleotide sequence and would likely amplify at a reduced efficiency with this primer-probe set.

**References**

1. Dabundo R, Lehmann MF, Treibergs L, Tobias CR, Altabet MA, Moisander PH, et al. The Contamination of Commercial ^15^N2 Gas Stocks with ^15^N-Labeled Nitrate and Ammonium and Consequences for Nitrogen Fixation Measurements. PLOS ONE. 2014;9: e110335. doi:10.1371/journal.pone.0110335

2. Weiss RF. The solubility of nitrogen, oxygen and argon in water and seawater. Deep Sea Research and Oceanographic Abstracts. 1970.

3. Church MJ, Jenkins BD, Karl DM, Zehr JP. Vertical distributions of nitrogen-fixing phylotypes at Stn ALOHA in the oligotrophic North Pacific Ocean. Aquat Microb Ecol. 2005;38: 3–14. doi:10.3354/ame038003

4. Church MJ, Short CM, Jenkins BD, Karl DM, Zehr JP. Temporal Patterns of Nitrogenase Gene (*nifH*) Expression in the Oligotrophic North Pacific Ocean. Appl Environ Microbiol. 2005;71: 5362–5370. doi:10.1128/AEM.71.9.5362-5370.2005

5. Moisander PH, Beinart RA, Hewson I, White AE, Johnson KS, Carlson CA, et al. Unicellular cyanobacterial distributions broaden the oceanic N2 fixation domain. Science. 2010;327: 1512–1514. doi:10.1126/science.1185468

6. Moisander PH, Beinart RA, Voss M, Zehr JP. Diversity and abundance of diazotrophic microorganisms in the South China Sea during intermonsoon. ISME J. 2008;2: 954–967. doi:10.1038/ismej.2008.51
