# Supplementary material for: Mesopelagic N2 Fixation Related to Organic Matter Composition in the Solomon and Bismarck Seas (Southwest Pacific)
Source: PLoS One. 2015 Dec 11;10(12):e0143775. doi: 10.1371/journal.pone.0143775 (PMC4684240; doi:10.1371/journal.pone.0143775)
Supplement: S1 Results — (DOCX) [file pone.0143775.s005.docx]

**S2 Results**

Hydrographic features and inorganic nutrients

The temperature in Transect 1 over the range of sampled depths (mesopelagic zone) was >15ºC above ~300 m, ~8-12ºC between 300-600 m, and ~5 between 600-1000 m (Supplementary Figure S1a). The distribution of temperatures was similar in Transect 2, although >15ºC waters were found down to ~300 m at the center of this transect (S1 Fig.). With the aim of finding a compromise between the range of temperatures measured in the mesopelagic zone, the ^15^N_2_-amended bottles were incubated in a temperature-controlled room set at 8ºC (see Methods). The temperature and salinity patterns co-varied. In Transect 1, salinities between ~34.8 and 35.2 were observed above ~300 m, followed by waters with a decreased salinity between 34.6 and 34.8 down to 500 m, and finally from ~500 to 1000 m the salinity was homogeneous at 34.5 (S1 Fig.). In agreement with the higher temperatures measured on Transect 2, higher salinities where measured in the waters above ~300 m (>35.3), while the rest of the water column had similar trends as Transect 1 (S1 Fig.).

The concentration of inorganic nutrients at the shallowest depth sampled (i.e. 250 m) was higher in Transect 1 than in Transect 2 (S1 Fig.), roughly following the distribution of temperature (S1 Fig.). In Transect 1, nutrient concentrations ranged between ~15 and 25 µM NO_x_ and ~1.2 to 1.8 µM PO_4_^3-^ at 250 m, while at the same depth, these concentrations were slightly lower in Transect 2 (10 µM NO_x_ and 1 µM PO_4_^3-^). Below 500-600 m however, 30-40 µM NO_x_ and ~2.3-3 µM PO_4_^3-^ were observed in both transects (S1 Fig.).

Bacterial counts

Bacterial abundances ranged from ~2.5 to 150 x 10^3^ cells mL^-1^, being generally higher between 250 and 600 m (S2 Fig.). In Transect 1, higher abundances were found at the western end of the transect, close to the Papua New Guinea coast (S2 Fig.). In Transect 2 there was no obvious association of higher bacterial abundance with the island coasts and the distribution was relatively homogeneous from 250 to 600 m (S2 Fig.). Equal abundances of ~0.5 x 10^5^ cells mL^-1^ were observed at stations 9 and 11 between 600 and 900 m in Transect 2 (S2 Fig.). Bacteria abundances were negatively correlated with inorganic nutrient concentrations (NO_x_ and PO_4_^3-^, both p < 0.0001), and hence positively correlated with temperature and salinity (both p < 0.0001).
