# Supplementary material for: Mesopelagic N2 Fixation Related to Organic Matter Composition in the Solomon and Bismarck Seas (Southwest Pacific)
Source: PLoS One. 2015 Dec 11;10(12):e0143775. doi: 10.1371/journal.pone.0143775 (PMC4684240; doi:10.1371/journal.pone.0143775)
Supplement: S1 Table — (DOCX) [file pone.0143775.s006.docx]

**Table S1:** *In situ* parameters measured at process stations where DOM addition experiments were performed.

| Station | Date | Depth (m) | Temperature (ºC) | Temperature of cold room (ºC) | Dissolved oxygen (mL L^-1^) | Salinity |
| --- | --- | --- | --- | --- | --- | --- |
| P1 | 7 March 2014 | 400 | 10.66 | 10-11 | 3.79 | 34.80 |
| P2 | 14 March 2014 | 300 | 12.16 | 12-13 | 3.76 | 34.92 |
| P3 | 20 March 2014 | 300 | 8.47 | 9-10 | 3.69 | 34.95 |
| P4 | 28 March 2014 | 300 | 13.75 | 13-14 | 1.60 | 35.07 |
