# Supplementary material for: Mesopelagic N2 Fixation Related to Organic Matter Composition in the Solomon and Bismarck Seas (Southwest Pacific)
Source: PLoS One. 2015 Dec 11;10(12):e0143775. doi: 10.1371/journal.pone.0143775 (PMC4684240; doi:10.1371/journal.pone.0143775)
Supplement: S2 Table — (DOCX) [file pone.0143775.s007.docx]

**Table S2:** qPCR primers and TaqMan probes designed for this study.

| Target clone | Closest *nifH* phylotype or Cluster | Forward primer (5’🡪3’) | Reverse primer (5’🡪3’) | Probe (5’🡪3’) |
| --- | --- | --- | --- | --- |
| M6411A02 | Deltaproteobacteria (Cluster III) | AGCCGGGTGTTGGTTGTG | CCGTGTAAGCGCCCTCTTC | TGGTGTTATCACAGCGATCAACTTCCTCG |
| M6413A02 | Cluster III | ACGAAGAAGATCAACAACTCGACTAC | GATCGGCATGGCAAATCC | CCTTTTACGACGTACTCGGCGACGTG |
| M6433A04 | Gammaproteobacteria (Cluster I) | CCGGGCGTCGGATGT | TCTTCGTAAGCACCTTCTTCTTCA | CCGTGGTGTCATTACCGCGATTAACTTTC |
