# Supplementary material for: Mesopelagic N2 Fixation Related to Organic Matter Composition in the Solomon and Bismarck Seas (Southwest Pacific)
Source: PLoS One. 2015 Dec 11;10(12):e0143775. doi: 10.1371/journal.pone.0143775 (PMC4684240; doi:10.1371/journal.pone.0143775)
Supplement: S3 Table — (DOCX) [file pone.0143775.s008.docx]

**Table S3:** List of clones and their corresponding GenBank accession number, phylotype and cluster (see Figure 8).

| Clone ID | Transect station | Depth (m) | Accession number | Phylotype | Cluster |
| --- | --- | --- | --- | --- | --- |
| M6411A01 | 1 | 740 | KT025938 | N7 | Alpha- and Betaproteobacteria |
| M6411A02 | 1 | 740 | KT025939 | N6 | Deltaproteobacteria |
| M6411A03 | 1 | 740 | KT025940 | N10 | Cluster III |
| M6411A04 | 1 | 740 | KT025941 | N7 | Alpha- and Betaproteobacteria |
| M6411A05 | 1 | 740 | KT025942 | N10 | Cluster III |
| M6411A06 | 1 | 740 | KT025943 | N7 | Alpha- and Betaproteobacteria |
| M6413A01 | 1 | 360 | KT025944 | N10 | Cluster III |
| M6413A02 | 1 | 360 | KT025945 | N10 | Cluster III |
| M6413A03 | 1 | 360 | KT025946 | N10 | Cluster III |
| M6413A04 | 1 | 360 | KT025947 | N10 | Cluster III |
| M6414A01 | 1 | 250 | KT025948 | N13 | Cluster III |
| M6414A02 | 1 | 250 | KT025949 | N10 | Cluster III |
| M6414A03 | 1 | 250 | KT025950 | N13 | Cluster III |
| M6414A04 | 1 | 250 | KT025951 | N10 | Cluster III |
| M6414A05 | 1 | 250 | KT025952 | N13 | Cluster III |
| M6422A01 | 3 | 300 | KT025953 | N10 | Cluster III |
| M6422A02 | 3 | 300 | KT025954 | N10 | Cluster III |
| M6422A03 | 3 | 300 | KT025955 | N10 | Cluster III |
| M6433A01 | 6 | 600 | KT025956 | N4 | Gammaproteobacteria |
| M6433A02 | 6 | 600 | KT025957 | N4 | Gammaproteobacteria |
| M6433A03 | 6 | 600 | KT025958 | N9 | Alpha- and Betaproteobacteria |
| M6434A01 | 6 | 350 | KT025959 | N3 | Gammaproteobacteria |
| M6434A02 | 6 | 350 | KT025960 | N4 | Gammaproteobacteria |
| M6434A03 | 6 | 350 | KT025961 | N5 | Gammaproteobacteria |
| M6434A04 | 6 | 350 | KT025962 | N4 | Gammaproteobacteria |
| M6434A05 | 6 | 350 | KT025963 | N3 | Gammaproteobacteria |
| M6437A01 | 7 | 400 | KT025964 | N6 | Deltaproteobacteria |
| M6437A02 | 7 | 400 | KT025965 | N6 | Deltaproteobacteria |
| M6437A03 | 7 | 400 | KT025966 | N6 | Deltaproteobacteria |
| M6437A04 | 7 | 400 | KT025967 | N11 | Cluster III |
| M6437A05 | 7 | 400 | KT025968 | N11 | Cluster III |
| M6412A01 | 1 | 250 | KT025969 | N14 | Cluster IV |
| M6412A02 | 1 | 250 | KT025970 | N8 | Alpha- and Betaproteobacteria |
| M6432A01 | 6 | 850 | KT025971 | N1 | Cyanobacteria |
| M6432A02 | 6 | 850 | KT025972 | N1 | Cyanobacteria |
| M6432A03 | 6 | 850 | KT025973 | N10 | Cluster III |
| M6432A04 | 6 | 850 | KT025974 | N1 | Cyanobacteria |
| M6432A05 | 6 | 850 | KT025975 |  | *Not included in phylogenetic tree |
| M6432A06 | 6 | 850 | KT025976 | N1 | Cyanobacteria |
| M6432A07 | 6 | 850 | KT025977 | N1 | Cyanobacteria |
| M6436A01 | 7 | 600 | KT025978 | N2 | Gammaproteobacteria |
| M6436A02 | 7 | 600 | KT025979 | N2 | Gammaproteobacteria |
| M6436A04 | 7 | 600 | KT025980 | N2 | Gammaproteobacteria |
| M6436A05 | 7 | 600 | KT025981 | N11 | Cluster III |
| M6436A06 | 7 | 600 | KT025982 | N2 | Gammaproteobacteria |
| M6439A01 | 8 | 900 | KT025983 | N1 | Cyanobacteria |
| M6439A02 | 8 | 900 | KT025984 | N1 | Cyanobacteria |
| M6439A03 | 8 | 900 | KT025985 | N1 | Cyanobacteria |
| M6439A04 | 8 | 900 | KT025986 | N1 | Cyanobacteria |
| M6439A05 | 8 | 900 | KT025987 | N1 | Cyanobacteria |
| M6439A06 | 8 | 900 | KT025988 | N1 | Cyanobacteria |
| M6439A07 | 8 | 900 | KT025989 | N12 | Cluster III |
| M6439A08 | 8 | 900 | KT025990 | N1 | Cyanobacteria |
| M6439A09 | 8 | 900 | KT025991 | N1 | Cyanobacteria |
| M6443A01 | 9 | 900 | KT025992 | N10 | Cluster III |
| M6443A02 | 9 | 900 | KT025993 | N10 | Cluster III |
| M6443A03 | 9 | 900 | KT025994 | N1 | Cyanobacteria |
| M6443A04 | 9 | 900 | KT025995 | N1 | Cyanobacteria |
| M6443A05 | 9 | 900 | KT025996 | N10 | Cluster III |
| M6443A06 | 9 | 900 | KT025997 | N1 | Cyanobacteria |
| M6443A07 | 9 | 900 | KT025998 | N10 | Cluster III |
| M6443A09 | 9 | 900 | KT025999 | N10 | Cluster III |
| M6444A01 | 9 | 600 | KT026000 | N1 | Cyanobacteria |
| M6444A02 | 9 | 600 | KT026001 | N1 | Cyanobacteria |
| M6444A03 | 9 | 600 | KT026002 | N1 | Cyanobacteria |
| M6444A04 | 9 | 600 | KT026003 | N1 | Cyanobacteria |
| M6444A05 | 9 | 600 | KT026004 | N1 | Cyanobacteria |
| M6445A01 | 9 | 420 | KT026005 | N2 | Gammaproteobacteria |
| M6445A02 | 9 | 420 | KT026006 | N2 | Gammaproteobacteria |
| M6445A03 | 9 | 420 | KT026007 | N2 | Gammaproteobacteria |
| M6445A04 | 9 | 420 | KT026008 | N10 | Cluster III |
| M6445A05 | 9 | 420 | KT026009 | N10 | Cluster III |
| M6445A06 | 9 | 420 | KT026010 | N1 | Cyanobacteria |
| M6445A07 | 9 | 420 | KT026011 | N2 | Gammaproteobacteria |
| M6445A08 | 9 | 420 | KT026012 | N1 | Cyanobacteria |
| M6445A09 | 9 | 420 | KT026013 | N10 | Cluster III |
| M6451A01 | 11 | 900 | KT026014 | N1 | Cyanobacteria |
| M6451A02 | 11 | 900 | KT026015 | N7 | Alpha- and Betaproteobacteria |
| M6451A03 | 11 | 900 | KT026016 | N1 | Cyanobacteria |
| M6451A04 | 11 | 900 | KT026017 | N1 | Cyanobacteria |
| M6451A05 | 11 | 900 | KT026018 | N1 | Cyanobacteria |
| M6451A06 | 11 | 900 | KT026019 | N1 | Cyanobacteria |
| M6451A07 | 11 | 900 | KT026020 | N1 | Cyanobacteria |
| M6451A08 | 11 | 900 | KT026021 | N7 | Alpha- and Betaproteobacteria |
| M6451A09 | 11 | 900 | KT026022 | N7 | Alpha- and Betaproteobacteria |
| M6453A01 | 11 | 400 | KT026023 | N1 | Cyanobacteria |
| M6453A02 | 11 | 400 | KT026024 | N1 | Cyanobacteria |
| M6453A03 | 11 | 400 | KT026025 | N1 | Cyanobacteria |
| M6453A04 | 11 | 400 | KT026026 | N1 | Cyanobacteria |
| M6453A05 | 11 | 400 | KT026027 | N1 | Cyanobacteria |
| M6453A06 | 11 | 400 | KT026028 | N1 | Cyanobacteria |
| M6453A07 | 11 | 400 | KT026029 | N1 | Cyanobacteria |
| M6454A01 | 11 | 280 | KT026030 | N1 | Cyanobacteria |
| M6454A02 | 11 | 280 | KT026031 | N10 | Cluster III |
| M6454A03 | 11 | 280 | KT026032 | N1 | Cyanobacteria |
| M6454A04 | 11 | 280 | KT026033 | N1 | Cyanobacteria |
| M6454A05 | 11 | 280 | KT026034 | N1 | Cyanobacteria |
