# Supplementary material for: Mesopelagic N2 Fixation Related to Organic Matter Composition in the Solomon and Bismarck Seas (Southwest Pacific)
Source: PLoS One. 2015 Dec 11;10(12):e0143775. doi: 10.1371/journal.pone.0143775 (PMC4684240; doi:10.1371/journal.pone.0143775)
Supplement: S4 Table — (DOCX) [file pone.0143775.s009.docx]

**Table S4:** Pearson correlation coefficients among hydrographic, chemical and biological variables, as well as the first four coordinate scores derived from principal coordinate analysis (PCoA) of dissolved organic matter (DOM) compounds relative peak intensities.

|  |  | Temp | Sal | O_2_ | NOx | PO_4_ | TEP | Bact | N_2_ fix | POC | N-org | P-org | Sat FA | Sat FA-X | Sugars | Sugars -X | Peptides | Coord 1 | Coord 2 | Coord 3 |
| --- | --- | --- | --- | --- | --- | --- | --- | --- | --- | --- | --- | --- | --- | --- | --- | --- | --- | --- | --- | --- |
| Temp | Pearson | 1 |  |  |  |  |  |  |  |  |  |  |  |  |  |  |  |  |  |  |
|  | Sig. |  |  |  |  |  |  |  |  |  |  |  |  |  |  |  |  |  |  |  |
|  | N | 48 |  |  |  |  |  |  |  |  |  |  |  |  |  |  |  |  |  |  |
| Sal | Pearson | ,964** |  |  |  |  |  |  |  |  |  |  |  |  |  |  |  |  |  |  |
|  | Sig. | ,000 |  |  |  |  |  |  |  |  |  |  |  |  |  |  |  |  |  |  |
|  | N | 48 |  |  |  |  |  |  |  |  |  |  |  |  |  |  |  |  |  |  |
| O_2_ | Pearson | ,059 | -,130 |  |  |  |  |  |  |  |  |  |  |  |  |  |  |  |  |  |
|  | Sig. | ,690 | ,377 |  |  |  |  |  |  |  |  |  |  |  |  |  |  |  |  |  |
|  | N | 48 | 48 |  |  |  |  |  |  |  |  |  |  |  |  |  |  |  |  |  |
| NOx | Pearson | -,961** | -,891** | -,267 |  |  |  |  |  |  |  |  |  |  |  |  |  |  |  |  |
|  | Sig. | ,000 | ,000 | ,067 |  |  |  |  |  |  |  |  |  |  |  |  |  |  |  |  |
|  | N | 48 | 48 | 48 |  |  |  |  |  |  |  |  |  |  |  |  |  |  |  |  |
| PO_4_ | Pearson | -,943** | -,838** | -,353* | ,985** |  |  |  |  |  |  |  |  |  |  |  |  |  |  |  |
|  | Sig. | ,000 | ,000 | ,014 | ,000 |  |  |  |  |  |  |  |  |  |  |  |  |  |  |  |
|  | N | 48 | 48 | 48 | 48 |  |  |  |  |  |  |  |  |  |  |  |  |  |  |  |
| TEP | Pearson | -,087 | -,123 | -,274 | ,122 | ,102 |  |  |  |  |  |  |  |  |  |  |  |  |  |  |
|  | Sig. | ,586 | ,443 | ,083 | ,446 | ,526 |  |  |  |  |  |  |  |  |  |  |  |  |  |  |
|  | N | 41 | 41 | 41 | 41 | 41 |  |  |  |  |  |  |  |  |  |  |  |  |  |  |
| Bact | Pearson | ,730** | ,715** | -,049 | -,668** | -,649** | -,192 |  |  |  |  |  |  |  |  |  |  |  |  |  |
|  | Sig. | ,000 | ,000 | ,748 | ,000 | ,000 | ,236 |  |  |  |  |  |  |  |  |  |  |  |  |  |
|  | N | 46 | 46 | 46 | 46 | 46 | 40 |  |  |  |  |  |  |  |  |  |  |  |  |  |
| N_2_ fix | Pearson | ,048 | ,008 | -,428** | ,067 | ,022 | ,382* | ,005 |  |  |  |  |  |  |  |  |  |  |  |  |
|  | Sig. | ,755 | ,961 | ,004 | ,666 | ,889 | ,020 | ,976 |  |  |  |  |  |  |  |  |  |  |  |  |
|  | N | 44 | 44 | 44 | 44 | 44 | 37 | 42 |  |  |  |  |  |  |  |  |  |  |  |  |
| POC | Pearson | -,007 | -,016 | -,099 | ,021 | ,034 | -,087 | ,007 | ,171 |  |  |  |  |  |  |  |  |  |  |  |
|  | Sig. | ,961 | ,912 | ,504 | ,889 | ,819 | ,589 | ,964 | ,266 |  |  |  |  |  |  |  |  |  |  |  |
|  | N | 48 | 48 | 48 | 48 | 48 | 41 | 46 | 44 |  |  |  |  |  |  |  |  |  |  |  |
| N-org | Pearson | -,231 | -,247 | -,178 | ,248 | ,242 | ,372 | -,451* | ,217 | ,333 |  |  |  |  |  |  |  |  |  |  |
|  | Sig. | ,289 | ,255 | ,416 | ,253 | ,266 | ,089 | ,035 | ,332 | ,121 |  |  |  |  |  |  |  |  |  |  |
|  | N | 23 | 23 | 23 | 23 | 23 | 22 | 22 | 22 | 23 |  |  |  |  |  |  |  |  |  |  |
| P-org | Pearson | -,540** | -,506* | -,256 | ,545** | ,542** | ,285 | -,682** | ,277 | ,221 | ,863** |  |  |  |  |  |  |  |  |  |
|  | Sig. | ,008 | ,014 | ,239 | ,007 | ,008 | ,199 | ,000 | ,211 | ,311 | ,000 |  |  |  |  |  |  |  |  |  |
|  | N | 23 | 23 | 23 | 23 | 23 | 22 | 22 | 22 | 23 | 23 |  |  |  |  |  |  |  |  |  |
| Sat FA | Pearson | -,174 | -,163 | -,216 | ,219 | ,205 | ,078 | -,064 | ,405 | -,128 | -,045 | ,060 |  |  |  |  |  |  |  |  |
|  | Sig. | ,426 | ,459 | ,322 | ,316 | ,347 | ,729 | ,776 | ,062 | ,560 | ,840 | ,786 |  |  |  |  |  |  |  |  |
|  | N | 23 | 23 | 23 | 23 | 23 | 22 | 22 | 22 | 23 | 23 | 23 |  |  |  |  |  |  |  |  |
| Sat FA-X | Pearson | -,295 | -,228 | ,088 | ,250 | ,264 | -,077 | -,203 | -,371 | ,180 | -,152 | ,040 | ,330 |  |  |  |  |  |  |  |
|  | Sig. | ,171 | ,296 | ,689 | ,249 | ,224 | ,734 | ,366 | ,089 | ,412 | ,487 | ,855 | ,124 |  |  |  |  |  |  |  |
|  | N | 23 | 23 | 23 | 23 | 23 | 22 | 22 | 22 | 23 | 23 | 23 | 23 |  |  |  |  |  |  |  |
| Sugars | Pearson | -,165 | -,175 | ,306 | ,017 | ,032 | ,082 | -,207 | -,544** | ,297 | ,110 | ,109 | -,358 | ,463* |  |  |  |  |  |  |
|  | Sig. | ,453 | ,423 | ,155 | ,939 | ,886 | ,716 | ,356 | ,009 | ,169 | ,616 | ,621 | ,094 | ,026 |  |  |  |  |  |  |
|  | N | 23 | 23 | 23 | 23 | 23 | 22 | 22 | 22 | 23 | 23 | 23 | 23 | 23 |  |  |  |  |  |  |
| Sugars -X | Pearson | ,254 | ,197 | -,054 | -,239 | -,241 | -,115 | ,246 | ,351 | -,127 | ,144 | -,019 | -,202 | -,888** | -,469* |  |  |  |  |  |
|  | Sig. | ,242 | ,368 | ,806 | ,273 | ,268 | ,612 | ,269 | ,109 | ,564 | ,512 | ,932 | ,355 | ,000 | ,024 |  |  |  |  |  |
|  | N | 23 | 23 | 23 | 23 | 23 | 22 | 22 | 22 | 23 | 23 | 23 | 23 | 23 | 23 |  |  |  |  |  |
| Peptides | Pearson | -,273 | -,198 | ,153 | ,223 | ,235 | -,150 | -,166 | -,480* | ,157 | -,192 | ,014 | ,264 | ,970** | ,468* | -,860** |  |  |  |  |
|  | Sig. | ,207 | ,364 | ,484 | ,307 | ,281 | ,505 | ,460 | ,024 | ,474 | ,381 | ,950 | ,223 | ,000 | ,024 | ,000 |  |  |  |  |
|  | N | 23 | 23 | 23 | 23 | 23 | 22 | 22 | 22 | 23 | 23 | 23 | 23 | 23 | 23 | 23 |  |  |  |  |
| Coord 1 | Pearson | -,564** | -,572** | -,138 | ,544** | ,524* | ,180 | -,735** | ,224 | ,288 | ,812** | ,787** | ,044 | -,078 | ,082 | ,097 | -,128 |  |  |  |
|  | Sig. | ,005 | ,004 | ,529 | ,007 | ,010 | ,423 | ,000 | ,316 | ,183 | ,000 | ,000 | ,840 | ,724 | ,709 | ,661 | ,561 |  |  |  |
|  | N | 23 | 23 | 23 | 23 | 23 | 22 | 22 | 22 | 23 | 23 | 23 | 23 | 23 | 23 | 23 | 23 |  |  |  |
| Coord 2 | Pearson | ,046 | ,082 | -,456* | ,037 | ,081 | ,631** | ,078 | ,648** | -,101 | ,306 | ,312 | ,010 | -,256 | -,143 | ,178 | -,286 | ,000 |  |  |
|  | Sig. | ,835 | ,711 | ,029 | ,865 | ,713 | ,002 | ,730 | ,001 | ,646 | ,156 | ,147 | ,965 | ,239 | ,515 | ,416 | ,185 | 1,000 |  |  |
|  | N | 23 | 23 | 23 | 23 | 23 | 22 | 22 | 22 | 23 | 23 | 23 | 23 | 23 | 23 | 23 | 23 | 23 |  |  |
| Coord 3 | Pearson | ,715** | ,658** | ,219 | -,685** | -,692** | -,073 | ,512* | -,121 | ,224 | ,366 | ,019 | -,123 | -,233 | -,079 | ,256 | -,208 | ,000 | ,000 |  |
|  | Sig. | ,000 | ,001 | ,315 | ,000 | ,000 | ,748 | ,015 | ,591 | ,304 | ,086 | ,930 | ,575 | ,284 | ,719 | ,238 | ,342 | 1,000 | 1,000 |  |
|  | N | 23 | 23 | 23 | 23 | 23 | 22 | 22 | 22 | 23 | 23 | 23 | 23 | 23 | 23 | 23 | 23 | 23 | 23 |  |
| Coord 4 | Pearson | ,262 | ,155 | ,329 | -,347 | -,383 | ,119 | ,007 | ,132 | -,564** | -,142 | -,256 | -,164 | -,293 | -,055 | ,158 | -,308 | ,000 | ,000 | ,000 |
|  | Sig. | ,227 | ,480 | ,125 | ,105 | ,071 | ,599 | ,975 | ,558 | ,005 | ,518 | ,239 | ,454 | ,174 | ,803 | ,473 | ,153 | 1,000 | 1,000 | 1,000 |
|  | N | 23 | 23 | 23 | 23 | 23 | 22 | 22 | 22 | 23 | 23 | 23 | 23 | 23 | 23 | 23 | 23 | 23 | 23 | 23 |

**Correlation is significant at the 0.01 level (2-tailed).

*Correlation is significant at the 0.05 level (2-tailed).
